# New Red-Emitting Chloride-Sensitive Fluorescent Protein with Biological Uses

**DOI:** 10.1021/acssensors.1c00094

**Published:** 2021-06-21

**Authors:** Rafael Salto, Maria D. Giron, Virginia Puente-Muñoz, Jose D. Vilchez, Laura Espinar-Barranco, Javier Valverde-Pozo, Daniele Arosio, Jose M. Paredes

**Affiliations:** † Department of Biochemistry and Molecular Biology II, Faculty of Pharmacy, Unidad de Excelencia en Quimica Aplicada a Biomedicina y Medioambiente (UEQ), University of Granada, Cartuja Campus, 18071 Granada, Spain; ‡ Department of Physical Chemistry, Faculty of Pharmacy, Unidad de Excelencia en Quimica Aplicada a Biomedicina y Medioambiente (UEQ), University of Granada, C. U. Cartuja, 18071 Granada, Spain; § Consiglio Nazionale delle Ricerche (CNR), Istituto di Biofisica (IBF-CNR), 38123 Trento, Italy

**Keywords:** red fluorescent proteins, chloride sensors, imaging, two-photon excitation microscopy, genetically encoded sensors, intracellular sensors

## Abstract

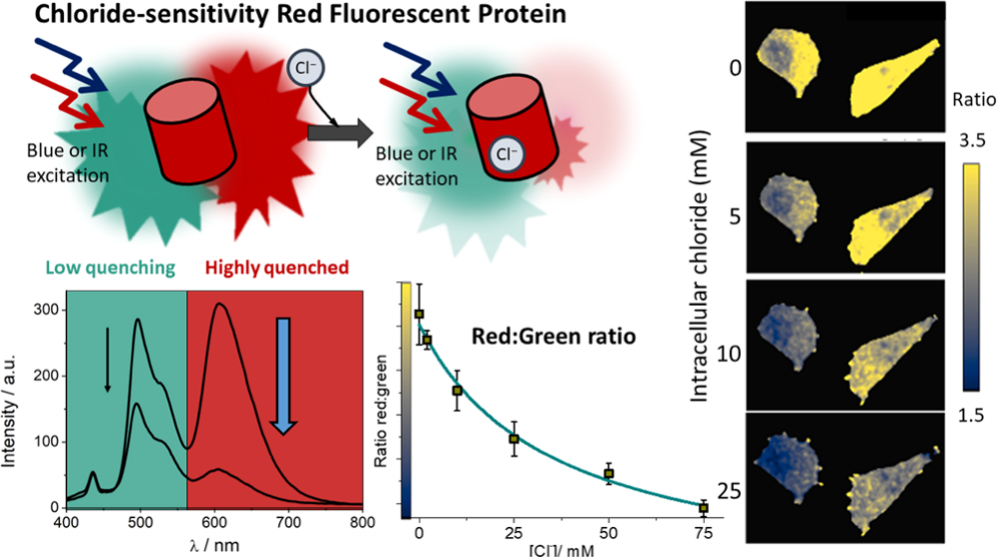

A new chloride-sensitive red fluorescent protein derived from *Entacmaea quadricolor* is described. We found that mBeRFP exhibited moderate sensitivity to chloride and, via site-directed mutagenesis (S94V and R205Y), we increased the chloride affinity by more than an order of magnitude (*k*_d_ = 106 ± 6 mM) at physiological pH. In addition, *cis–trans* isomerization of the chromophore produces a dual emission band with different chloride sensitivities, which allowed us to develop a ratiometric methodology to measure intracellular chloride concentrations.

The flow of different ions between the intracellular and extracellular media has key functions in maintaining cellular homeostasis. Among them, chloride, as the most abundant physiological anion, has important roles in processes^1^ such as volume regulation,^2^ membrane potential,^3^ and neuroexcitation.^4^ In addition, chloride concentration regulates intraorganelle functions, for example, in mitochondria^5^ and endosomes,^6^ and is even involved in control of the expression of specific genes.^7^ Chloride homeostasis alterations are the basis of many human diseases,^8^ such as cystic fibrosis (CF), the most lethal genetic disease affecting Caucasians.^9^ CF is caused by dysfunction in the cystic fibrosis transmembrane conductance regulator (CFTR) transporter, whose activity is currently assayed by monitoring chloride flow through the channel.^10^

In recent decades, the importance of chloride in physiological and pathological processes has promoted the development of intracellular probes to monitor the flow of chloride in living cells. However, this task presents multiple difficulties and challenges.^11^ In this regard, noninvasive experimental techniques combined with imaging techniques have proven to be extraordinary tools for monitoring chloride concentrations in living cells. To that end, probes of different natures, from organic fluorescent dyes to recently developed DNA nanodevices, have been used.^12,13^ Among them, since the discovery of the chloride sensitivity of yellow fluorescent protein (YFP), fluorescent proteins have had an important role.^14^ Since then, fluorescent proteins have been developed to act as chloride sensors due to the possibility of modifying their primary structure to generate new mutants with improved chloride sensitivity^15^ via sensor engineering.^16−21^ The mechanism of all these proteins is based on fluorescence quenching; however, recently, a natural, chloride-sensitive, turn-on, yellow fluorescent protein has been discovered.^22,23^

To date, efforts in the development of new fluorescent chloride sensors have been based on mutations of the YFP or green fluorescent protein (GFP),^15^ but the insertion of chloride-binding domains into fluorescent proteins of different colors remains to be attempted. Of particular interest would be the generation of chloride-sensitive moieties in red- and infrared-emitting proteins. Through the use of two-photon excitation,^24^ chloride-sensitive red proteins would open new opportunities for chloride measurement imaging because redshifted signals exhibit less autofluorescence, deeper tissue penetration, and less light scattering.

In this work, we present a new family of chloride-sensitive red fluorescent proteins derived from *Entacmaea quadricolor*.^25^ Our precursor is an improved long Stokes shift (LSS) red fluorescent protein, termed mBeRFP, that is derived from mKate.^26^ LSSmKate2 was used for the development of LSSmClopHensor, which simplified the use of ClopHensor and extended its utility for two-photon excitation microscopy.^24^ Although LSSmKate2 exhibits pH- and chloride-independent fluorescence emission, we noticed small responses to chloride at very low pH values. Chloride binding is energetically favored at low pH values because the electrostatic repulsion exerted by the anionic chromophore is nearly absent.^27^ When the pH is below the p*K*_a_, the prototropic chromophore form is nearly absent and chloride affinity is high. So, mBeRFP caught our attention being a variant of LSSmKate2 with a remarkably higher p*K*_a_ value of 5.6. However, the effects of chloride binding on mBeRFP fluorescence emission have not been studied. The presence of a chloride sensing domain in mBeRFP is a very interesting feature with multiple potential uses in bioimaging as a chloride sensor. To the best of our knowledge, this is the first observation of the presence of a chloride-sensing motif in a fluorescent protein family beyond the green/yellow proteins.

## Experimental Section

### Site-Directed Mutagenesis and Cloning

The plasmid pRSET B-BeRFP, which codes for the mBeRFP protein,^26^ was kindly provided by Dr. Zhihong Zhang (Britton Chance Center for Biomedical Photonics, Wuhan National Laboratory for Optoelectronics, Huazhong University of Science and Technology, Wuhan, Hubei, China). The bacterial expression plasmid pET21-LSSmKate2, which encodes the LSSmKate2 fluorescent protein, has been described elsewhere.^21^ To clone the BeRFP coding sequence into a eukaryotic expression vector, PCR amplification of the coding sequence from the plasmid pRSET B-BeRFP was carried out using the oligonucleotides BeRFP_F_ and BeRFP_R_ (see the Supporting Information (SI), Table S1), which include a Kozac sequence plus an *Age*I restriction site and a *Bgl*II restriction site, respectively. The PCR product was cloned into the pJET 1.2 vector (Thermo Fisher Scientific, Madrid, Spain) and then digested with *Age*I–*Bgl*II to replace the eGFP coding sequence in the pEGFP-C1 eukaryotic expression vector (Clontech Laboratories, Palo Alto, CA). The new plasmid was termed pBeRFP-C1.

Site-directed mutagenesis of either the pRSET B-BeRFP or pBeRFP-C1 plasmid was carried out as described previously^28^ to introduce mutations in the BeRFP coding sequence. The oligonucleotides used for site-directed mutagenesis are provided in the SI, Table S1. The sequences of all of the generated plasmids were confirmed by automatic sequencing with universal primers.

### Recombinant Protein Expression and Purification

For bacterial expression of BeRFP and its mutated versions, BL21(pLys) competent cells were transformed with the pRSET B-BeRFP plasmid (or the mutated plasmids) and grown in a Luria*–*Bertani (LB) broth with selected antibiotics at 37 °C overnight without the addition of an inductor, and bacterial pellets were obtained by centrifugation. For the expression of LSSmKate2, BL21(pLys) bacteria transformed with the pET21b-LSSmKate2 plasmid were grown in LB broth with selected antibiotics at 37 °C until reaching an OD_600 nm_ = 0.5. Then, the bacteria were induced with 1 mM IPTG and further grown overnight at 37 °C. Bacterial pellets were obtained by centrifugation.

For purification of the proteins, bacterial pellets were resuspended in the dilution buffer (20 mM HEPES, 20 mM imidazole, 500 mM NaCl, pH 8) containing 1 mM PMSF and 2 μg/mL aprotinin and sonicated. The lysed bacterial suspension was centrifuged for 30 min at 12 000*g* to remove the cell debris, and the supernatant was filtered through a 0.45 μm filter. A HisTrap FF crude 1 mL column (GE Healthcare Life Sciences, Chicago, IL) was equilibrated in the dilution buffer, and the supernatant was then loaded on the column. After washing, the proteins were eluted using 20 mM HEPES and 500 mM imidazole, pH 8. The eluted proteins were concentrated and dialyzed against PBS, and the protein concentration was measured using the bicinchoninic acid (BCA) method. Protein purity was confirmed by SDS-PAGE.

### Bioinformatics Design of 3D Structures

Modeling of the mutants was performed using Dynamut software,^29^ and the crystal structure of LSSmKate (PBD ID: 3NT3) was used as a starting point. Additionally, the figures showing 3D structures were constructed using the site-directed mutagenesis wizard tools of Swiss-PdbViewer (Swiss Institute of Bioinformatics) and pyMol.

### Spectroscopy Analysis of Proteins *In Vitro*

Absorption spectra were obtained on a Lambda 650 UV–visible spectrophotometer (PerkinElmer, Waltham, MA). Fluorescence excitation/emission spectra were collected on a Jasco FP-8300 spectrofluorimeter (Jasco, Tokyo, Japan).

The p*K*_a_ values were determined by fitting the maximum intensity (*I*) at different pH values using the following equation
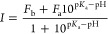
1where *F*_a_ and *F*_b_ are the plateau values for fluorescence intensity under acidic and basic conditions, respectively.

Chloride binding was studied by selecting the maxima of fluorescence emission while titrating the fluorescent protein at different chloride concentrations. The ionic strength was kept constant at 0.5 M by the addition of Na_2_SO_4_. The fluorescence intensity (*I*) was fitted using the following 1:1 binding equation
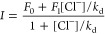
2where *k*_d_ is the dissociation constant, [Cl^–^] is the chloride concentration, and *F*_0_ and *F*_1_ are the fluorescence signals at zero and infinite chloride concentrations, respectively. Curve fitting was performed using Origin 8.5 (OriginLab). The chloride dependence on pH was studied, revealing the cooperative binding of chloride and protons via the following expression
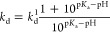
3where *k*_d_^1^ is the chloride affinity of the proton-ligated form of mBeRFP S94V-R205Y and p*K*_a_ is the logarithm of the association constant of protons in the absence of chloride. Curve fitting was performed using Origin 8.5 (OriginLab).

Quantum yield calculations were performed by measuring both the absorbance and fluorescence of fluorescent protein solutions in TRIS buffer using mBeRFP as a reference at pH = 7.30. For the relative determination of the fluorescence quantum yield (Φ), the following formula was used
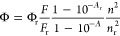
4Here, the subscript r refers to the reference (mBeRFP),^26^*F* is the integrated fluorescence spectrum, *A* denotes the absorbance at the excitation wavelength used, and *n* represents the refractive index of the solvent.

Time-resolved fluorescence decay traces were collected via the time-correlated single-photon counting (TCSPC) method using a FluoTime 200 fluorometer (PicoQuant, GmbH). The sample was excited using a 375 nm laser pulse at a frequency of 20 MHz. The fluorescence emission was focused at the detector after crossing through a polarizer (set at the magic angle), 2 mm slits, and a 2 nm bandwidth monochromator. The TCSPC was measured at 36 ps/channel. Fluorescence decay traces were collected to reach 20 000 counts at the peak channel. The emission wavelengths were recorded at 495 and 610 nm. The fluorescence decay traces were fitted to two- and three-exponential functions using a deconvolution method (FluoFit 4.4 package, Picoquant GmbH). For each sample, the decay traces were fitted globally with the decay times linked as shared parameters, whereas the pre-exponential factors were locally adjustable parameters.

### Fluorescence Microscopy and Image Analysis

Images of the fluorescence emission intensities were recorded on a MicroTime 200 fluorescence microscope system (PicoQuant GmbH, Berlin, Germany). The excitation source consisted of a pulsed diode laser (LDH series from PicoQuant) at λ = 375 nm. The light beam was directed onto a dichroic mirror (DCXR 375 nm, AHF-Chroma) and to the oil immersion objective (100×, 1.4 NA) of an inverted microscope system (IX-71, Olympus, Tokyo, Japan). The fluorescence emission was directed to a 405 nm longpass filter (AHF LP 405 nm, AHF Semrock) and focused on a 75 μm pinhole. The fluorescence was then passed through a dichroic filter (dcx600; AHF/Chroma), and the fluorescence was split into two different single-photon avalanche diodes (SPCM-AQR 14, PerkinElmer) through two bandpass filters: 630/60 (red channel) (Thorlabs) and 520/35 (green channel) (Thorlabs). Imaging reconstruction, photon counting, and data acquisition were realized with a TimeHarp 200 TCSPC module (PicoQuant, Berlin, Germany). Raw 512 × 512 pixel images were obtained. Two-photon imaging was performed using a Leica SPS II confocal microscope equipped with a Mai Tai Multiphoton laser (λ = 750 nm). The objective used was a PL APO 63×/1.2 CS water immersion device. Fluorescence was acquired using a hybrid detector (HyD, Leica).

Image processing was performed using custom Fiji macros. After importing the raw images, a Gaussian smoothing function was applied (s.d. = 0.5, in pixels). The region of interest (ROI) was manually selected to create binary masks: 0 for background and 1 for cells. Both channels were multiplied by the ROI to obtain images that only contained the values for the cells. Finally, the images were divided to obtain the ratiometric image.

### Cell Culture and DNA Transfection Assays

Human embryonic kidney 293 (HEK-293; ECACC 85120602), human colorectal adenocarcinoma (Caco2; ECACC 86010202), and murine neuroblastoma Neuro-2a (N2a; ATCC: CCL-131) cells were supplied by the Cell Culture Facility (University of Granada, Spain). Cells were grown at 37 °C in Dulbecco’s modified Eagle’s medium (DMEM) supplemented with 10% (v/v) fetal bovine serum (FBS), 2 mM glutamine, 100 U/mL penicillin, and 0.1 mg/mL streptomycin.

Before transfection, cells were seeded onto coverslips in 6-well plates at a density of 2.3 × 10^5^ cells/well for 24 h to reach a cell confluence of 80–90%. For transfection experiments, pmBeRFP and pmBeRFP-S94V-R205Y plasmids (4.3 μg/well) were mixed with LP2000 (10 μL) at room temperature for 30 min in a final volume of 100 μL. Next, the mixture was diluted to 1 mL with DMEM without FBS and added to each well. Cells were incubated with the polyplexes for 5 h. The transfection medium was then removed, and cells were further grown in DMEM plus 10% FBS for an additional period of 24 h. Transfected cells were used for fluorescence microscopy and image analysis.

In the N2a cell proliferation experiments, DMEM supplemented with 10% FBS was used. For differentiation experiments, the medium was replaced with DMEM supplemented with 1% FBS.

### Cell Permeabilization

Cells were seeded onto coverslips in 6-well plates and incubated in their respective medium. On the day of the experiment, the cells were washed with phosphate-buffered saline (PBS) and perforated by incubation for 15 min at 37 °C with 2 μg/mL α-toxin in a permeabilization buffer (20 mM potassium MOPS, pH 7.0, 250 mM mannitol, 1 mM potassium ATP, 3 mM MgCl_2_, and 5 mM potassium glutathione). Afterward, the cells were washed with the corresponding buffer three times and analyzed via fluorescence microscopy.

## Results and Discussion

### Rational Mutagenesis and Photophysics

**Figure 1:**
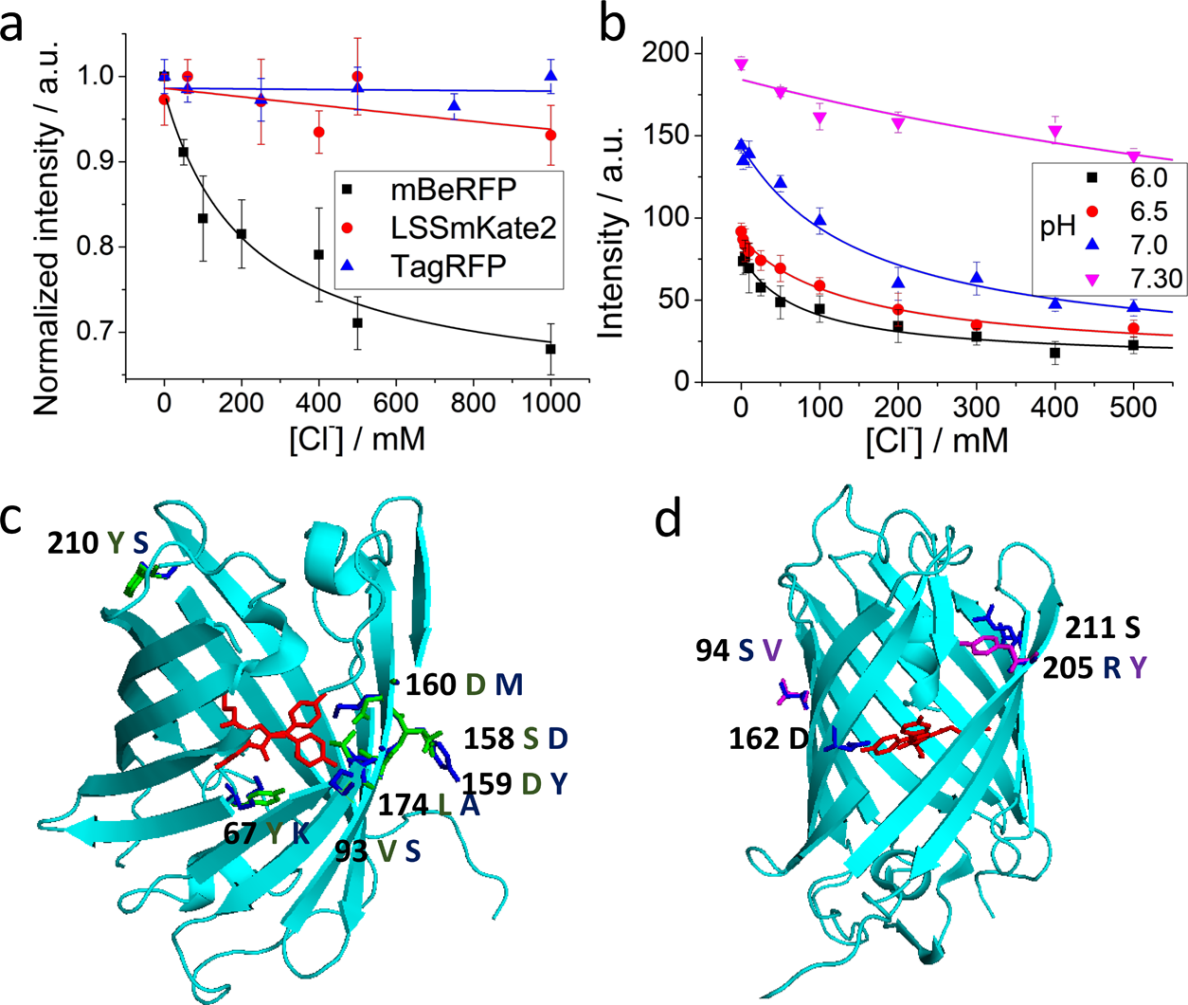
Influence of chloride on the fluorescence intensity of mBeRFP and its precursors. (a) Dependence of the fluorescence intensities of LSSmKate2, TagRFP, and mBeRFP on the chloride concentration at pH = 7.35. Excitation was set at a maximum excitation wavelength (440 nm for LSSmKate2 and mBeRFP and 585 nm for TagRFP), and emission was recorded at 610 nm. Bars represent the standard error obtained from 3 replicates. (b) Dependence of the mBeRFP fluorescence intensity on the chloride concentration at four different pH values. Excitation was set at 440 nm, and emission was recorded at 610 nm. Bars represent the standard error obtained from 3 replicates. (c) View of the amino acid positions mutated in LSSmKate2 (green, first position amino acid letter) and mBeRFP (blue, second position amino acid letter). The chromophore is highlighted in red. Three-dimensional conformations were calculated using Dynamut software, and the crystal structure of LSSmKate (PBD ID: 3NT3) was used as a starting point. (d) View of the amino acid positions additionally mutated in mBeRFP (blue, first position amino acid letter) in this study: S94V and R205Y (purple, second position amino acid letter), and other amino acids mentioned.

Since mBeRFP precursors, such as LSSmKate2 and TagRFP, do not show detectable chloride sensitivity (Figure 1a) at physiological pH values, we started trying to elucidate the molecular basis for the chloride sensitivity of mBeRFP compared with LssmKate2. The chloride affinity of mBeRFP is extremely low at physiological pH: 1380 mM at pH 7.3, and 78 mM at pH 6.0 (Figure 1b and Table 1), and this prompted us to mutate certain key amino acids to increase the sensitivity of mBeRFP to chloride. mBeRFP presents five^26^ and seven^30^ mutations compared to its precursors mKate and LSSmKate2, respectively. Since the structure of mBeRFP has not been experimentally determined, we modeled it based on the known spatial conformation of LssmKate2 (PBD ID: 3NT3). For this purpose, the analysis software (Dynamut) that allows the prediction of protein stability changes upon mutation^29^ was used to model the structural changes produced by the Y71K, V97S, S162D, D163Y, D164M, L178A, and Y214S mutations present in mBeRFP (amino acid residues are numbered throughout the manuscript according to the mBeRFP protein as indicated in the SI, Figure S1. In LssmKate2, amino acids 3–6 are missing, and therefore, numeration in LSSmKate2 is shifted by 4 positions. In LSSmKate2, the excited-state proton transfer (ESPT) reaction is responsible for the LSS character, and Asp160 is the last amino acid in the proton pathway that interacts with the chromophore.^30^ The equivalent amino acid in mBeRFP is Asp162,^26^ and from the model (Figure 1c), it can be proposed that Asp162 is the amino acid responsible for the LSS character of mBeRFP due to an excited-state proton transfer reaction^30^ and could also be involved in chloride sensitivity. LSSmKate2 has a Ser162 residue, and to demonstrate the relevance of position 162 in chloride sensitivity in mBeRFP, we mutated this Asp to Ser, Ala, or Thr (Table 1 and SI, Table S2). The oligonucleotide sequences are reported in the SI, Table S1).

**Table 1:** Chloride Sensitivity of mBeRFP and Mutants at Position 162^a^

	LSS	p*K*_a_	*k*_d_/mM (pH = 6.0)	*k*_d_/mM (pH = 6.5)	*k*_d_/mM (pH = 7.0)	*k*_d_/mM (pH = 7.3)
mBeRFP	yes	5.98 ± 0.14	78 ± 22	145 ± 33	161 ± 15	1380 ± 270
D162S	no	5.81 ± 0.18	24.5 ± 7.0	54 ± 9.5	77 ± 25	416 ± 69
D162A	no	5.38 ± 0.05	–	1060	–	–
D162T	no	5.51 ± 0.08	–	insensitive	–	–

a Dashes indicate conditions not measured.

In mBeRFP, reversion of Asp162 to Ser improves the affinity for chloride, demonstrating the relevance of this position to chloride sensing. Therefore, a smaller radical that retains a protonatable group, such as Ser, improves the chloride affinity. Because the precursor proteins mKate and LSSmKate-2 have a Ser in this position, the question arises whether they both would potentially be sensitive to chloride. However, since LSSmKate-2 has a very low p*K*_a_ (2.5), the chromophore is deprotonated even at acidic pH, and its electrochemical interactions with the negatively charged chloride anion hinder binding and make the fluorescence of the protein insensitive to chloride. As mKate has a higher p*K*_a_ (6.2), further research is needed to study the presence or absence of the chloride binding site in this RFP.

Mutations in this position to other amino acids, such as Ala or Thr, resulted in decreased sensitivity to chloride. This is likely due to the loss of charge when Ala is inserted, preventing the formation of hydrogen bonds, or to the greater steric hindrance caused by the methyl group of Thr.

The pH dependence of chloride affinity is recognized as a critical challenge in chloride detection for many chloride-sensing fluorescent proteins. In YFP-based chloride sensors, such as Cl^–^ monomeric YFP,^31^ the Ser203 side-chain is responsible for this pH dependence. This Ser mediates proton transfer from the external solvent to the internal chromophore,^31^ and incorporation of Val in this position in this yellow protein blocks the proton transfer pathway, which increases chloride affinity and decreases the pH dependence.^31^

Following a similar strategy, in mBeRFP, there are two Ser residues (at positions 94 and 211) with the ability to mediate proton transfer to the chromophore. Of these, in our structural model, only the Ser94 side chain is oriented toward the chromophore (Figure 1d and the SI). Therefore, site-directed mutagenesis was carried out at Ser94, and its mutation to Val strongly increased the affinity for chloride at the four measured pH values (Table 2). We investigated the dependence of the fluorescence intensity spectrum on pH and chloride, and the spectra are shown in the SI, Figures S2 and S3, respectively. Moreover, this mutation causes the fluorescence intensity of the protein pH-independent, although its affinity for chloride is still pH-dependent.

**Table 2:** Chloride Sensitivity of mBeRFP and Mutants^a^ at Positions 94, 162, and 205

	LSS	p*K*_a_	*K*_d_/mM (pH = 6.0)	*K*_d_/mM (pH = 6.5)	*K*_d_/mM (pH = 7.0)	*K*_d_/mM (pH = 7.3)
mBeRFP	yes	5.98 ± 0.14	78 ± 22	145 ± 33	154 ± 20	1380 ± 270
S94V	yes	4.20 ± 0.52	11.2 ± 3.0	21.7 ± 5.5	70 ± 27	793 ± 145
R20SY	yes	5.59 ± 0.61	5.19 ± 0.63	27.8 ± 2.6	98.8 ± 14.8	125 ± 13
D162S-S94V	no	6.12 ± 0.09	21.1 ± 7.5	77 ± 18	173 ± 46	191 ± 47
S94V-R205Y	yes	6.13 ± 0.08	4.63 ± 0.34	21.7 ± 2.5	66 ± 13	106 ± 6
D162S-R205Y	no	7.52 ± 0.04	18.6 ± 3.1	51.3 ± 6.7	136 ± 28	189 ± 44
D162S-S94V-R205Y	no	7.48 ± 0.03	21.7 ± 5.6	56.4 ± 7.3	145 ± 28	277 ± 47

a Effect of mutation on dissociation constants at four different pH values; LSS characteristics and p*K*_a_ values are shown.

Finally, in YFP and E^2^GFP, Tyr203 is essential for the presence of a chloride binding site.^14^ Therefore, since there is an Arg in the equivalent position in mBeRFP, we mutated it to Tyr (Table 2). Satisfactorily, this mutation led to a strong increase in the affinity for chloride. In the SI, Figure S4, we show the effect of chloride on the intensity at four different pH values for all the mutants studied in this work.

Since the mBeRFP side chains at 94, 162, and 205 are especially relevant for the chloride affinity of the protein, we analyzed combined mutations at these three positions to study the effects on chloride binding and pH dependence. The *K*_d_ values calculated for all the mutants are summarized in Table 2 and SI, Figure S5. The S94V-R205Y double mutation produced the highest affinity to chloride, even at physiological pH. In comparison, YFP-H148Q had a *K*_d_ = 154 mM, and only a few YFPs show a better *K*_d_ at physiological pH.^15^ Overall, our mBeRFP-S94V-R205Y mutant is a red fluorescent protein with sensitivity to chloride on par with YFP, and chloride-sensitive YFP variants engineered thus far. The proteins reported in this work can serve as a basis for the further development of chloride-sensitive RFPs.

Photophysical properties are key to ensure a high signal-to-noise ratio. Therefore, we measured the quantum yield and brightness for all mBeRFP variants examined; the results are listed in the SI, Table S3. The relative brightness of all mutants decreased in comparison with the original mBeRFP, with the exception of the single mutation D162S that retains almost the same value. However, these brightness values are enough to grant its usefulness as sensors in live cells.

**Figure 2:**
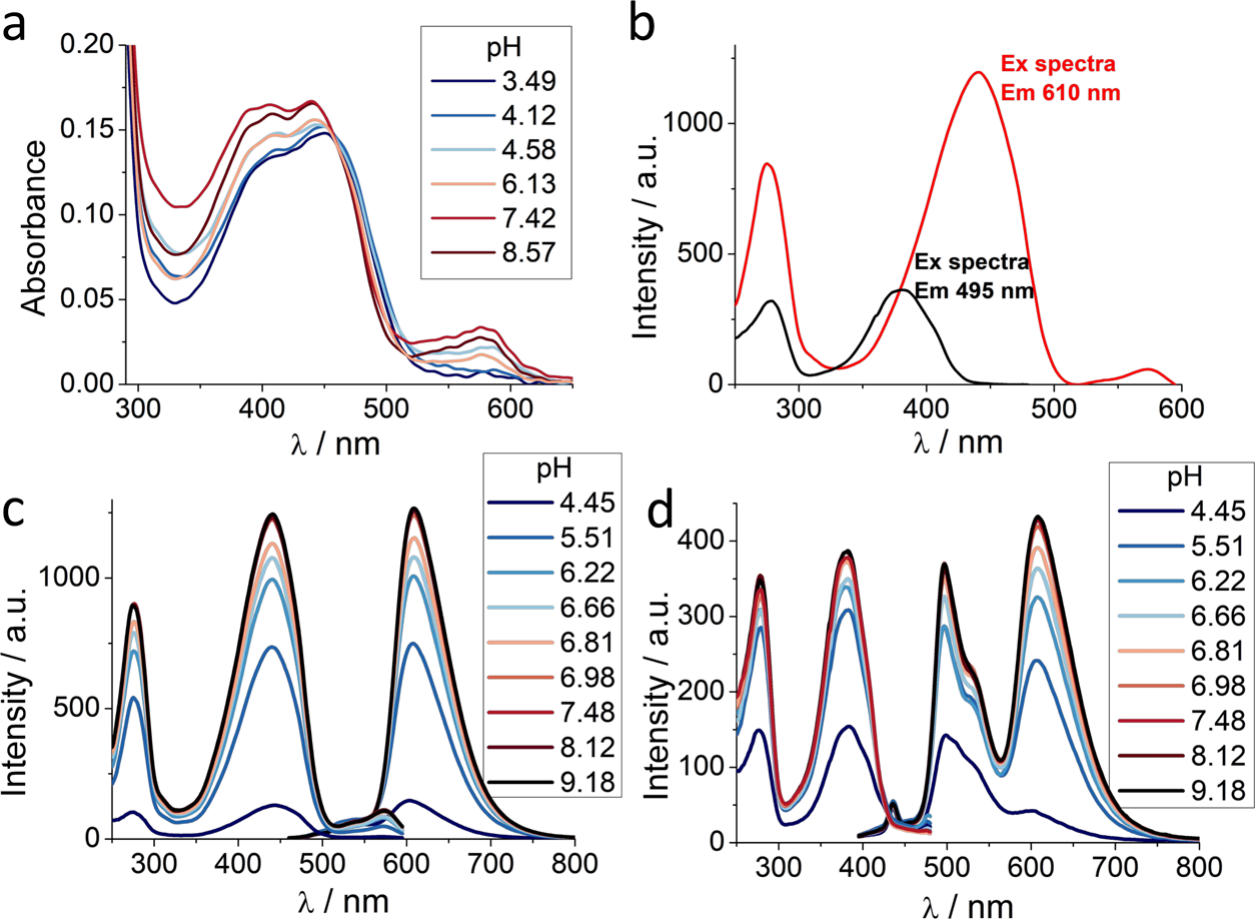
Influence of *cis–trans* isomerization on the spectral properties of mBeRFP and its derivatives. (a) Absorption spectra of mBeRFP at different pH values. (b) Excitation spectra of both emission maxima (λ emission: 495 nm (black) and 610 nm (red)). (c) Excitation (λ emission 610 nm) and emission (λ excitation: 440 nm) fluorescence spectra of mBeRFP S94V-R205Y at different pH values. (d) Excitation (λ emission: 495 nm) and emission fluorescence (λ excitation: 375 nm) spectra of mBeRFP S94V-R205Y at different pH values.

Another relevant aspect of the use of mBeRFP derivatives for chloride sensing is that the mBeRFP precursors (mKate and LssmKate2) exhibit *cis–trans* isomerization of the chromophore induced by pH.^32−34^ This isomerization generates different absorption and emission bands. Although this molecular mechanism is well established,^34^ to the best of our knowledge, the dual emission band from the equilibrium between both isomers has not been used in any biological application. In this work, we report for the first time that mBeRFP displays pH-dependent *cis–trans* isomerization with maximum absorption at 380 and 440 nm at low and high pH, respectively. Although it is difficult to observe isomerization in the absorption spectra (Figure 2a) due to the overlap of both bands, the excitation spectra (Figure 2b) clearly show both excitation/emission pairs. In this figure, it can be observed that the excitation band centered at 380 nm is responsible for the emission band centered at 495 nm and the excitation band centered at 440 nm is responsible for the emission centered at 610 nm. Figure S6 in the SI shows the signal ratio between both absorption and excitation bands, where at a pH above ∼7.5, there is only one isomer. In contrast, below this pH (including at physiological pH), both isomers are present. Therefore, due to the *cis*–*trans* isomerization of mBeRFP, two maximum emission bands are found at 495 and 610 nm at low and high pH, respectively. Although the red fluorescence emission (λ_em_ = 610 nm) can easily be isolated using excitation at 440 nm (see Figure 2c), at 380 nm, both absorption bands overlap, and thus, it is possible to excite simultaneously both isomers and recover the dual-band emission (see Figure 2d). Therefore, the presence of pH-dependent isomerization in the protein allows the determination of intracellular pH by measuring both fluorescence emission intensities and discriminating the pH (acidic or basic) in different intracellular compartments, such as lysosomes or mitochondria, respectively.

All the mutants generated from mBeRFP retained double-band emission owing to the *cis*–*trans* isomerization. We selected the mBeRFP S94V-R205Y mutant for further studies because of its higher affinity for chloride. Since the red isomers exhibited a redshift in the absorption band relative to the green isomers, the pH dependence of the fluorescence of the mBeRFP S94V-R205Y mutant was studied by selectively exciting the red isomer (Figure 2c) and performing simultaneous excitation of both isomers (Figure 2d). Due to the pH dependence of *cis*–*trans* isomerization, the green band is lower at high pH and higher at low pH in comparison with the red emission band.

**Figure 3:**
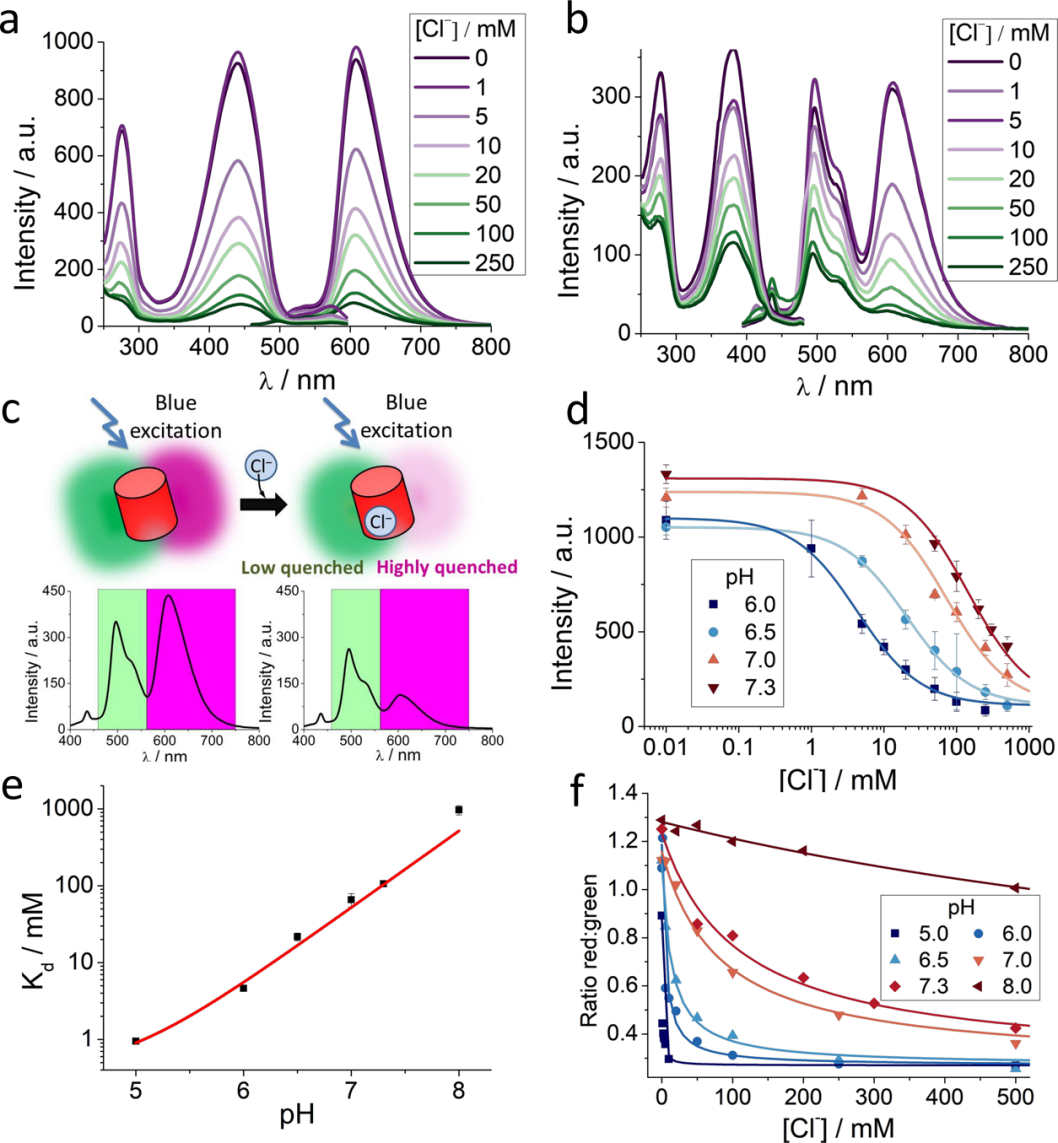
Influence of chloride on the spectral properties of mBeRFP and its derivatives. (a) Excitation (λ emission: 610 nm) and emission fluorescence spectra of mBeRFP S94V-R205Y with an excitation wavelength of 440 nm at different chloride concentrations and pH = 6.0. (b) Excitation (λ emission: 495 nm) and emission fluorescence spectra of mBeRFP S94V-R205Y with an excitation wavelength of 380 nm at different chloride concentrations and pH = 6.0. (c) Proposed scheme of the methodology of mBeRFP S94V-R205Y as a chloride biosensor. (d) Fluorescence intensity vs chloride concentration at four different pH values. Error bars represent the standard error obtained from three measurements. (e) Dependence of the dissociation constant on pH. The solid line was obtained by global data fitting to eq 3 with the following fit parameters: p*K*_a_ = 6.08 ± 0.30, and *K*_d_^1^ = 0.98 ± 0.17 mM. Error bars represent the standard error. (f) Dependence of the red/green fluorescence ratio on the chloride concentration at different pH values. Error bars represent the standard error obtained from three measurements.

Next and more relevant, we studied the effect of chloride on the green and red emission bands. Interestingly, the bands were affected differently by chloride. Although both emissions are quenched, this effect is significantly stronger under red fluorescence than under green emission (Figure 3a,b). This fact opens the possibility of using the red/green ratio to evaluate the concentration of chloride in samples (Figure 3c). Using this approach, we used only one excitation wavelength and recovered the fluorescence in two separate channels (green and red). The calibration of the red/green ratio dependence on chloride concentration allowed us to accurately measure the anion levels.

Similar to chloride-sensitive YFP/GFP,^27,35^ the chloride sensitivity of mBeRFP S94V-R205Y was affected by the environmental pH (Figure 3d,e and SI, Figure S7). This is an important issue that also occurs in the other chloride-sensitive fluorescent proteins described and can introduce a potential error source in chloride determination upon pH changes. For this reason, the use of mBeRFP S94V-R205Y as a chloride sensor should be limited to a pH-controlled environment, or mBeRFP S94V-R205Y should be used simultaneously with a pH indicator. In this case, the pH indicator should be spectrally compatible with mBeRFP S94V-R205Y. In future developments, this problem can be solved by developing constructs made of two-colored proteins to simultaneously detect pH and chloride concentration.^20,21^Figure 3f shows the change in the ratio in response to the chloride concentration at four different pH values.

It is worth noting that although there is an apparent modest change in the fluorescence intensity around physiological pH (see Figure 2c), there is a significant change in the affinity constant of chloride in this pH range (see Figure 3d,e). This is because the p*K*_a_ of this mutant is 6.13 ± 0.08, and therefore, in the pH range from 7.0 to 7.3, there is a predominant population of the anionic form of the chromophore. Despite the small changes observable in the fluorescence signal, in the physiological pH range, chloride binding remains strongly hindered by electrostatic repulsion stemming from the negative charge of the chromophore. Indeed, small variations in the pH elicit large variations in *K*_d_ for chloride in the physiological pH range (see Figure 3d–f).

To investigate the selectivity of this sensor to chloride, we measured the effect of different anions on fluorescence emission. As expected, anionic salts commonly used as buffers had no effect on the intensity (sulfate, phosphate, and TRIS). By contrast, halogens produced a quenching effect that was stronger with the radius of the ion (I^–^ > Br^–^ > Cl^–^ > F^–^) (see the SI, Figure S8), similar to that occurring in the GFP/YFP chloride sensors, thus confirming similar binding mechanisms. Static quenching via the formation of a complex between the fluorescent protein and the chloride ion was confirmed by measuring the fluorescence lifetime in the absence and in the presence of chloride (concentrations: 0 and 500 mM) at both emission wavelengths (495 and 610 nm). At 610 nm, the decay traces were fitted with a biexponential curve with recovered fluorescence lifetimes of 3.29 ± 0.02 and 2.17 ± 0.01 ns at 0 mM chloride. With the addition of 500 mM chloride, a third fluorescence lifetime of 0.33 ± 0.01 ns appeared. At 495 nm, the decay traces at both chloride concentrations (0 and 500 mM) were fitted with a triexponential curve with recovered fluorescence lifetimes of 3.18 ± 0.03, 1.43 ± 0.02, and 0.32 ± 0.01 ns.

### Intracellular Sensing

Genetically encoded fluorescent sensors are widely used to measure processes in cells and tissues; for this reason, one potential use of the new chloride-sensitive red fluorescent proteins should be to quantify chloride fluxes within cells. First, we determined the photobleaching effect in solutions and in cells, as can be observed in the SI, Figure S10. Although there is a small photobleaching effect in the solution, it is similar in both channels, and therefore, with the ratio calculations used, the effect is almost negligible. In HEK-293 cells, photobleaching only affects the ratio during the first 5 min producing a slight decrease but remaining constant after this time.

To determine the ability of mBeRFP and mBeRFP S94V-R205Y to detect intracellular chloride levels, HEK-293 cells were transfected with plasmids encoding these proteins. We selected a wavelength of 375 nm to excite both isomers and recovered the emission in two different channels (green and red) to obtain both emission bands. Similar to the solution analysis results, the relationship between the red and green channels should depend on the chloride concentration.

**Figure 4:**
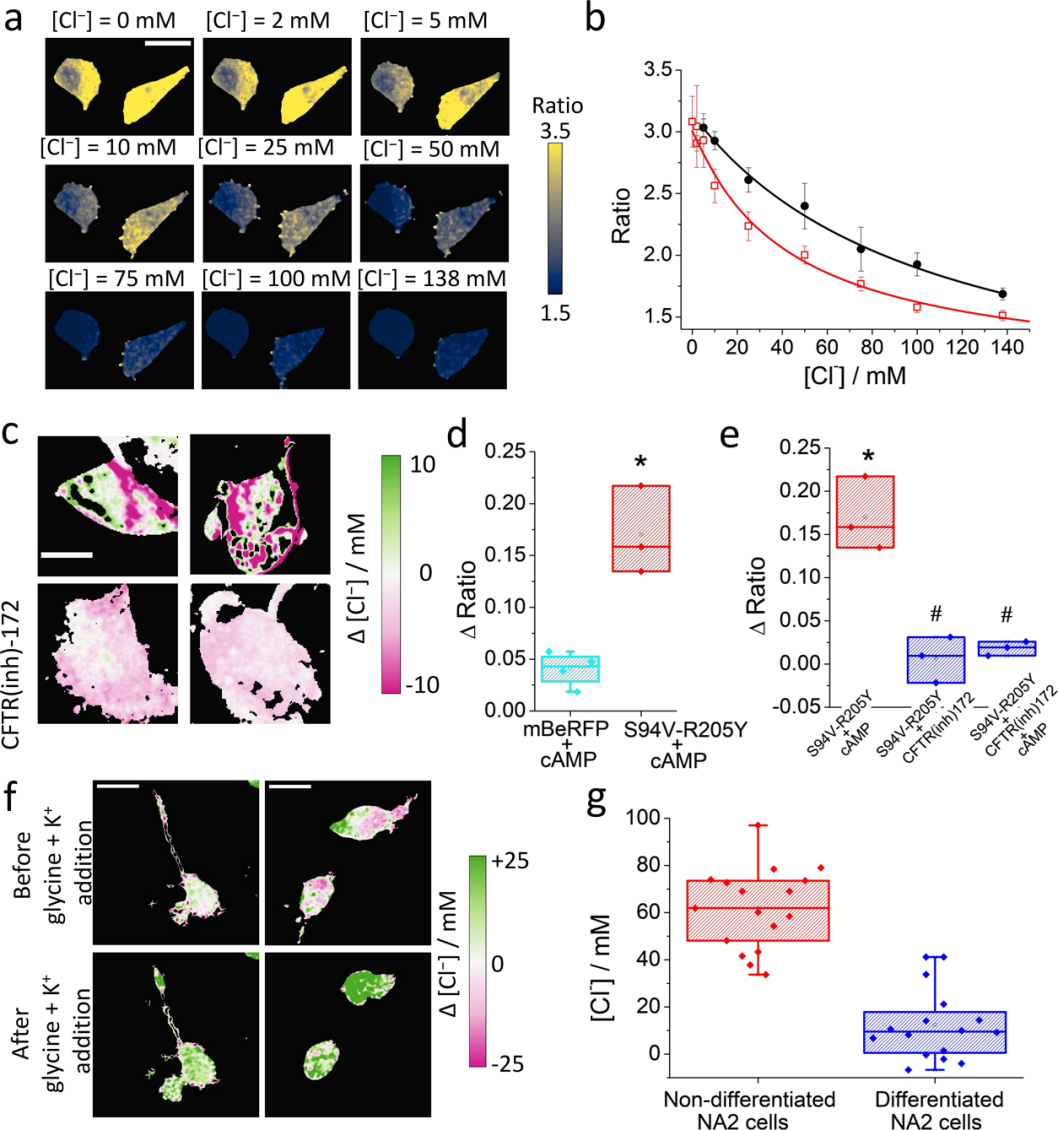
Live measurement of chloride concentrations using a modified mBeRFP protein. (a) Red/green ratio maps of HEK-293 cells clamped at different chloride concentrations. (b) Ratio changes in HEK-293 cells transfected with pmBeRFP (open squares) and pClopHensor (circles) clamped at different chloride concentrations. Fitting curves were drawn using eq 2 in the Experimental Section; scale bar: 20 μm. (c) Chloride changes in CaCo-2 cells transfected with pmBeRFP S94V-R205Y after the addition of 50 μM Br-cAMP to cells that were previously incubated in the absence (upper line) or presence (lower line) of the inhibitor CFTR(inh)-172 (10 μM) for 30 min; scale bar: 10 μm. (d) The Box and whisker plot representing the changes in the red/green ratio after incubation of CaCo-2 cells transfected with mBeRFP or mBeRFP S94V-R205Y and 50 μM Br-cAMP. (e) The Box and whisker plot representing the changes in the red/green ratio after incubation of CaCo-2 cells transfected with mBeRFP S94V-R205Y with 50 μM Br-cAMP in the absence or presence of 10 μM CFTR(inh)-172. The asterisks indicate a significant difference between the column and the mBeRFP population and a hash indicates a significant difference between the inhibited populations and the mBeRFP S94V-R205Y column based on a nonparametric statistical *t*-test at the 0.95 confidence level. (f) Effects of glycine and K^+^ on the intracellular chloride concentration in the N2a cell line. (g) The Box and whisker plot representing the average intracellular chloride concentration in nondifferentiated and differentiated N2a cells. Boxes correspond to 25 and 75%, with a horizontal bar indicating the average value. Whiskers represent the minimum and maximum values.

The sensitivity of mBRFP and mBeRFP S94V-R205Y to different chloride concentrations was verified by clamping the extracellular chloride concentration and incubating the cells with α-toxin from *Staphylococcus aureus*, a nonspecific ionophore, allowing chloride clamping between the extracellular and intracellular media, as has been well established in previous studies.^36^ Raw images and ratio maps of transfected cells are presented in Figure 4a, Figures S10–S13 and Movie S1 in the SI. In this experiment, some changes in the shape of the cells were observed due to the use of α-toxin and the time extension of these measurements, but these changes did not compromise the use of the sensor because the cells were clamped, with no effect on the fluorescence intensities of both channels and therefore without artifacts that preclude its use to determine the intracellular pH and chloride concentration. Upon increase of the intracellular chloride concentration, the red/green ratio showed a remarkable decrease for the mBeRFP S94V-R205Y mutant and not for the original mBeRFP (see the SI, Figures S13 and S14). The range of sensitivity to chloride of the mBeRFP S94V-R205Y mutant allowed for accurate measurements even at low chloride concentrations in the physiological pH range, with a fair overlap with the intracellular chloride concentration range expected in epithelial cells (40–110 mM).^37^ The intracellular sensitivity of mBeRFP S94V-R205Y in HEK-293 cells was measured following the same protocol previously described. As can be observed in Figure 4a, the ratio was dependent on the chloride concentration, and the ratio average is presented in Figure 4b (open squares). In this figure, the intracellular chloride affinity (*k*_d_ = 44 ± 8 mM) of mBeRFP S94V-R205Y is compared to the affinity of ClopHensor (Figure 4b, black circles, and SI, Figure S15, the ratio maps).

Cystic fibrosis is the most common autosomal-recessive genetic disease in humans.^38^ Mutations in the CFTR gene that encodes the CFTR protein, a chloride channel located in the apical membrane of exocrine epithelial cells, are the basis of this disorder, which is characterized by impaired physiological chloride flux. We verified the use of mBeRFP S94V-R205Y as a chloride biosensor in a simulated impaired CTFR using the inhibitor CFTR(inh)-172. For this purpose, we selected the CaCo-2 cell line, which is an epithelial cell line that expresses CFTR channels and is derived from colon adenocarcinoma in a Caucasian patient. CFTR is a cAMP-dependent ion channel whose activation is affected in cystic fibrosis cells. The addition of a nonhydrolyzable cAMP (Br-cAMP) (50 μM) for 30 min to the cell cultures produces a chloride flux from the intracellular to the extracellular medium. In Figure 4d, the differences in the ratio signals of four and three different cell images obtained before and after the addition of cAMP are reported. When mBeRFP S94V-R205Y**-**transfected cells were preincubated with the inhibitor CFTR(inh)-172 (10 μM) for 30 min, subsequent addition of Br-cAMP did not significantly alter the intensity of either channel, and the corresponding pale colors can be observed in Figure 4c (and Figure S16, SI), with minor modifications of the red/green ratio signal and therefore negligible changes in the intracellular chloride concentrations. As shown in Figure 4c, sections inside the cells presented a strong pink color, indicating a decrease in the intracellular chloride concentration corresponding to an increase in the ratio value.

Figure 4e summarizes the change in the fluorescence ratio in mBeRFP S94V-R205Y mutant cells after the addition of Br-cAMP to CaCo-2 cells and changes in the ratio of mBeRFP S94V-R205Y-expressing cells after the addition of the inhibitor and then Br-cAMP. These results demonstrate the usefulness of this new mutant as a new genetically encoded double green and red emission intracellular monomeric chloride sensor.

To further assess the sensitivity of this biosensor, we demonstrated its use in neuronal cellular systems. Our first approach was to measure changes in the intracellular chloride concentration in N2a cells through the cation-chloride cotransporter.^39^ We stimulated these receptors by adding glycine in combination with high K^+^ to the extracellular solution. This elicited an intracellular increase of 16 ± 9 mM chloride (see Figure 4f), in agreement with previously reported observations.^39^ Then, we measured the chloride concentration under physiological conditions in populations of N2a cells before and after differentiation. Our results showed a decrease in the chloride concentration when N2a cells were differentiated, as shown in Figure 4g (ratio maps can be observed in the SI, Figure S17). In the chloride maps in Figure 4, we found a large variation in the intracellular chloride, but given the diffusivity of the chloride ion, these intracellular gradients are likely artifacts of the imaging/measurement process.

**Figure 5:**
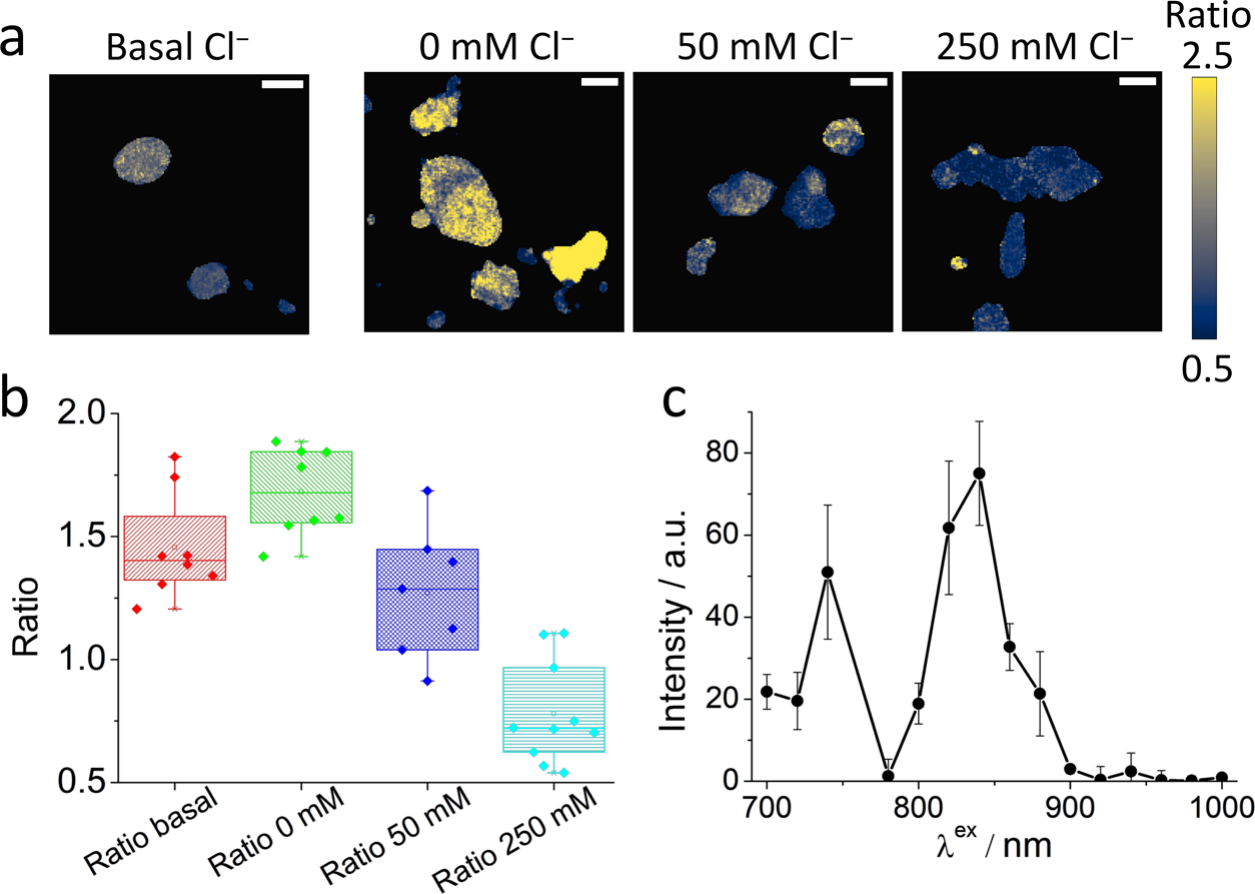
Two-photon excitation imaging measurements. (a) Red/green ratio maps of HEK-293 cells obtained using two-photon excitation (λ_ex_ = 750 nm). The figure shows images reflecting the basal chloride concentration (left) and images of cells clamped at different chloride concentrations; scale bars: 20 μm. (b) The Box and whisker plot representing the average red/green ratio from the two-photon excitation data. Boxes correspond to 25 and 75%, with the horizontal bar indicating the average value. Whiskers represent the minimum and maximum values. (c) Two-photon excitation spectra measured in HEK-293 cells transfected with mBeRFP S94V-R205Y. Emission collected at 600–650 nm, and excitation ranging from 700 to 1000 nm, measured every 20 nm. The intensities were calculated by normalization to the power of the excitation source measured on the excitation pathway. Bars represent the standard error from three replicates.

Finally, to assess new opportunities for chloride imaging in tissues, we explored the ability to perform a bioimaging analysis using infrared excitation (λ_ex_ = 750 nm) through two-photon absorption. Figure 5a shows the red/green ratio images of the basal chloride concentration and cells clamped at three different chloride concentrations (raw images are shown in the SI, Figure S18). The red/green ratio decreases when the intracellular chloride concentration is high. Basal cells show a chloride concentration of less than 50 mM. Figure 5b summarizes all of the recovered ratio values in a box and whisker plot, showing good chloride sensitivity using this approach in two-photon excitation images.

Finally, we acquired two-photon excitation spectra measuring the intensity achieved in HEK-293 cells after stimulation with excitation wavelengths ranging from 700 to 1000 nm. Our results show two peaks after two-photon excitation, one at approximately 750 nm and the other at approximately 840 nm (see Figure 5c). In this respect, for future uses in vivo, the main obstacle is the dependence of excitation and emission light propagation in tissues on the wavelength.^24^ This issue produces strong errors in the measurements, even in ratiometric assays. However, it is possible to correct this issue through an invariant reference. To extend its usefulness, mBeRFP S94V-R205Y might be linked with far-red, chloride-independent fluorescent proteins as an invariant reference.

Despite the pH-dependent chloride sensitivity, which is ingrained in all GFP- and YFP-based chloride sensors, our results clearly demonstrate the utility of mBeRFP S94V-R205Y in applications in cells. Future development for increasing the chloride affinity and reducing the pH dependence will be possible as already occurring for other chloride-sensitive GFPs and YFPs.^40^

Herein, we have presented a novel bright red fluorescent protein for the ratiometric measurement of chloride in living cells. The ratiometric signal originates from two spectrally distinct emission bands without requiring the addition of a second fluorescent protein. Therefore, mBeRFP S94V-R205Y holds great potential for physiology studies about dynamic changes in chloride that might occur in scenarios in which pH changes are not expected.

## Conclusions

To summarize, we have discovered a new family of chloride-sensitive proteins with dual-band emission. Through site-directed mutagenesis, we greatly improved the chloride affinity at physiological pH. Moreover, we developed and verified a new methodology using the red/green fluorescence ratio and one- or two-photon excitation to monitor the flow of chloride in cells, and using a specific CFTR inhibitor, cystic fibrosis conditions were simulated in the cell model. From a future perspective, these proteins can be used as the backbone of the next generation of chloride sensors, where the two following significant approaches can be employed to minimize the pH dependence of chloride sensitivity: (i) increasing the p*K*_a_ of the chloride biosensor to minimize its chloride dependence at physiological pH and (ii) developing new biosensors linking two different fluorescent proteins (a pH sensor fused to this chloride sensitivity protein) to measure the pH and chloride concentration. These developments will allow measuring the intracellular chloride in scenarios where a variation of pH is expected.

## Supporting Information Available

Alignment of FP variants; oligonucleotides used in this article; chloride titration at different pH values; chloride titration at four different pH values; relative quantum yields and brightnesses; mBeRFP S94V-R205Y anion dependence; mBeRFP S94V-R205Y photobleaching; raw images of HEK-293 cells transfected with the S94V-R205Y mutant obtained using one-photon excitation microscopy; representative ratio maps of ClopHensor calibration; chloride changes in CaCo-2 cells transfected with pmBeRFP S94V-R205Y; and ImageJ macro used to process and analyze the images obtained in this work (PDF)
Red/green ratio maps of HEK-293 cells transfected with mBeRFP or pmBeRFP S94V-R205Y and clamped at different chloride concentrations (MP4)
