# Supplementary material for: New Red-Emitting Chloride-Sensitive Fluorescent Protein with Biological Uses
Source: ACS Sens. 2021 Jun 21;6(7):2563–73. doi: 10.1021/acssensors.1c00094 (PMC8478333; doi:10.1021/acssensors.1c00094)
Supplement: Supplementary file 1 — se1c00094_si_001.pdf [file se1c00094_si_001.pdf]

Supplementary Information

New Red-Emitting Chloride-Sensitive Fluorescent Protein with Biological Uses

Rafael Salto[1], María Dolores Girón[1], Virginia Puente-Muñoz[2], José Dámaso Vílchez[1], Laura Espinar-Barranco[2], Javier Valverde-Pozo[2], Daniele Arosio[3,*], José Manuel Paredes[2,*]

[1] Department of Biochemistry and Molecular Biology II, Faculty of Pharmacy, Unidad de Excelencia en Quimica Aplicada a Biomedicina y Medioambiente (UEQ), University of Granada, Cartuja Campus, 18071 Granada, Spain.

[2] Department of Physical Chemistry, Faculty of Pharmacy, Unidad de Excelencia en Quimica Aplicada a Biomedicina y Medioambiente (UEQ), University of Granada, C. U. Cartuja, 18071 Granada, Spain.

[3] Consiglio Nazionale delle Ricerche (CNR). Istituto di Biofisica (IBF-CNR). Trento, Italy.

* Corresponding author 's email: daniele.arosio@cnr.it, jmparedes@ugr.es;

Figure S1. Alignment of FP variants.
Table S1. Oligonucleotides used in this article.
Table S2. Mutations introduced in the proteins compared to LSS-mKate2.
Figure S2. pH dependence of the fluorescence intensity of all the mutants studied in this work.
Figure S3. Chloride titration at different pH values.
Table S3. Relative quantum yields and brightnesses.
Figure S4. Chloride titration at four different pH values. Fitting curves.
Figure S5. pH dependence of the dissociation constant.
Table S3. *Relative quantum yields and brightnesses*.
Figure S6. *Cis/trans isomerization in the absorption and excitation ratio values.*
Figure S7. *Linkage between H^+ and Cl^- binding to mBeRFP S94V-R205Y.*
Figure S8. *mBeRFP S94V-R205Y anion dependence.*
Figure S9. *Fluorescence decay histograms of S94V-R205Y mBeRFP*
Figure S10. *mBeRFP S94V-R205Y photobleaching*.
Figure S11. Raw images of HEK-293 cells transfected with mBeRFP obtained using one photon-excitation microscopy.
Figure S12. Raw images of HEK-293 cells transfected with the S94V-R205Y mutant obtained using one-photon excitation microscopy.
Figure S13. Red/green ratio maps of HEK-293 cells clamped at different chloride concentrations.
Figure S14. Ratio changes in HEK-293 cells transfected with pmBeRFP or pmBeRFP S94V-R205Y and clamped at different chloride concentrations.
Figure S15. Representative ratio maps of ClopHensor calibration.
Figure S16. Chloride changes in CaCo-2 cells transfected with pmBeRFP S94V-R205Y.
Figure S17. Representative ratio maps of nondifferentiated and differentiated neuro 2a cells.
Figure S18. Raw images of HEK-293 cells transfected with the S94V-R205Y mutant obtained using two-photon excitation microscopy.
ImageJ macro used to process and analyze the images obtained in this work.
Video S1. Legend

```
       LSS-mKate2   1 MS----ELIKENMHMKLYMEGTVNNHHFKCTSEGEGKPYEGTQTMRIKVVEGGPLPFAFD  56
                      |:    ||||||||||||||||||||||||||||||||||||||||||||||||||||||
           mBeRFP   1 MVSKGEELIKENMHMKLYMEGTVNNHHFKCTSEGEGKPYEGTQTMRIKVVEGGPLPFAFD  60
                      ||||||||||||||||||||||||||||||||||||||||||||||||||||||||||||
mBeRFP S94V-R205Y   1 MVSKGEELIKENMHMKLYMEGTVNNHHFKCTSEGEGKPYEGTQTMRIKVVEGGPLPFAFD  60

       LSS-mKate2  57 ILATSFMYGSYTFINHTQGIPDFFKQSFPEGFTWERVTTYEDGGVLTATQDTSLQDGCLI  116
                      |||||||||| ||||||||||||||||||||||||| |||||||||||||||||||||||
           mBeRFP  61 ILATSFMYGSKTFINHTQGIPDFFKQSFPEGFTWERSTTYEDGGVLTATQDTSLQDGCLI  120
                      ||||||||||| ||||||||||||||||||||||||| ||||||||||||||||||||||
mBeRFP S94V-R205Y  61 ILATSFMYGSKTFINHTQGIPDFFKQSFPEGFTWERVTTYEDGGVLTATQDTSLQDGCLI  120

       LSS-mKate2 117 YNVKIRGVNFTSNGPVMQKKTLGWEAGTEMLYPADGGLEGRSDDALKLVGGGHLICNLKS  176
                      |||||||||| |||||||||||||||:||||||||||||||:::|||||||||||||:|
           mBeRFP 121 YNVKIRGVNFPSNGPVMQKKTLGWEASTEMLYPADGGLEGRDYMALKLVGGGHLICNAKT  180
                      ||||||||||||||||||||||||||||||||||||||||||||||||||||||||||||
mBeRFP S94V-R205Y 121 YNVKIRGVNFPSNGPVMQKKTLGWEASTEMLYPADGGLEGRDYMALKLVGGGHLICNAKT  180

       LSS-mKate2 177 TYRSKKPAKNLKVPGVYYVDRRLERIKEADKETYVEQHEVAVARYCDLPSKLGHKLN  233
                      ||||||||||||:|||||||||||||||||||| |||||||||||||||||||||
           mBeRFP 181 TYRSKKPAKNLKMPGVYYVDRRLERIKEADKETSVEQHEVAVARYCDLPSKLGHK--  235
                      |||||||||||||||||||||||| ||||||||||||||||||||||||||||||
mBeRFP S94V-R205Y 181 TYRSKKPAKNLKMPGVYYVDRRLEYIKEADKETSVEQHEVAVARYCDLPSKLGHK--  235
```

Figure S1. *Alignment of FP variants.* In the previously described sequence of mBERFP (https://www.fpbase.org/protein/mberfp/), the numbering is modified because the first two amino acids are numbered 1 and 1a. In this article, they are numbered 1 and 2.

Table S1. *Oligonucleotides used in this article*. Only the sequence of the forward oligonucleotide is shown for the site-directed mutagenesis reactions. For this technique, a reverse complementary oligonucleotide to the one shown was also used in the mutagenic PCR (1).

Name	Sequence	Restriction site	Purpose
BeRFP$_{D162S}$	5´-gac ggt gga ctg gaa ggc aga tcc tac atg gcc ctg aag ctc gtg ggc-3`<br>D G G L E G R S Y M A L K L V G	New *Hinc*II	Site-directed mutagenesis of BeRFP
BeRFP$_{D162A}$	5´-gac ggc gga ctg gaa ggc aga gcc tac atg gcc ctg aag ctc gtg ggc-3`<br>D G G L E G R A Y M A L K L V G	*Bgl*I Removal	Site-directed mutagenesis of BeRFP
BeRFP$_{D162T}$	5´-gac ggc gga ctg gaa ggc aga acc tac atg gcc ctg aag ctc gtg ggc-3`<br>D G G L E G R T Y M A L K L V G	*Bgl*I Removal	Site-directed mutagenesis of BeRFP
BeRFP$_{S94V}$	5´-gag ggc ttc aca tgg gag aga gtc acc aca tat gaa gac ggg-3´<br>E G F T W E R V T T Y E D G	New *Nde*I	Site-directed mutagenesis of BeRFP
BeRFP$_{R205Y}$	5´-gtg gac aga aga ctc gag tat atc aag gag gcc gac aaa gag-3´<br>V D R R L E Y I K E A D K E	New *Xho*I	Site-directed mutagenesis of BeRFP
BeRFP$_F$	5´-a ccg gtc gcc acc atg gtg tct aag ggc gaa gag ctg att aag-3´	New *Age*I	Cloning of BeRFP in pEGFP-C1
BeRFP$_R$	5´- aga tct gag tcc gga aag ttt gtg ccc cag ttt gct agg g-3´	New *Bgl*II	Cloning of BeRFP in pEGFP-C1

(1) Kucinska M, Giron MD, Piotrowska H, Lisiak N, Granig WH, Lopez-Jaramillo FJ, et al. Novel Promising Estrogenic Receptor Modulators: Cytotoxic and Estrogenic Activity of Benzanilides and Dithiobenzanilides. PloS one. 2016; 11: e0145615.

Table S2. *Mutations introduced in the proteins compared to LSS-mKate2.* The color code for mutated amino acids matches that used in Figure S1.

**LSS-mKate2**	**mBeRFP**	**mBeRFP S94V-R205Y**
Tyr67	Lys71	Lys71
Val93	Ser97	Val97
Ser158	Asp162	Asp162
Asp159	Tyr163	Tyr163
Asp160	Met164	Met164
Leu174	Ala178	Ala178
Tyr210	Ser214	Ser 214
Arg 201	Arg 205	Tyr 205

mBeRFP

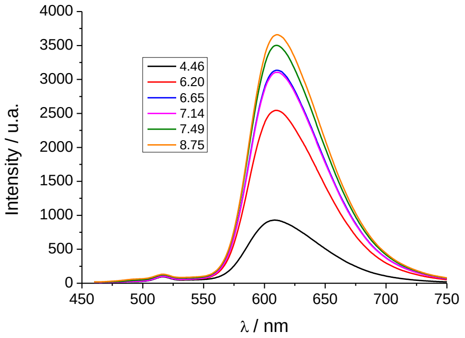

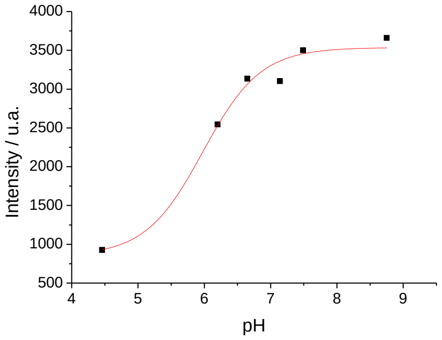

D162S

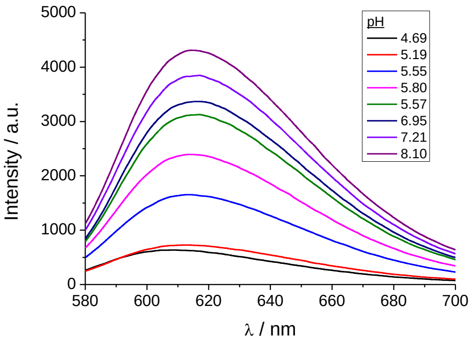

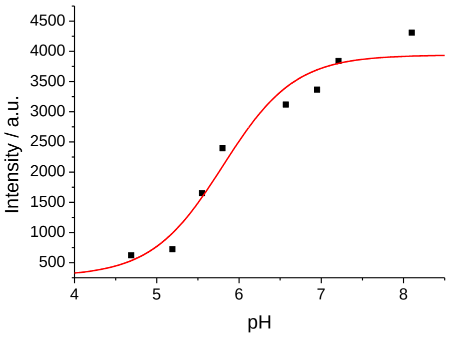

D162A

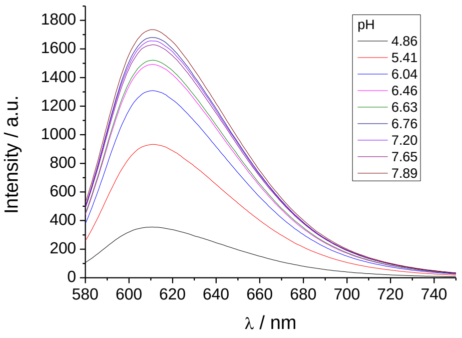

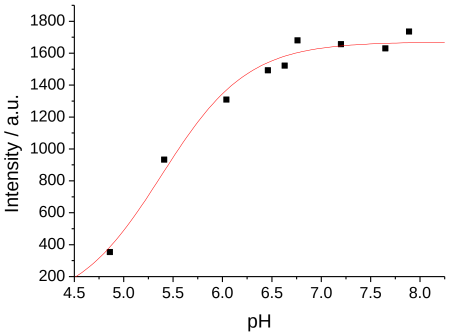

D162T

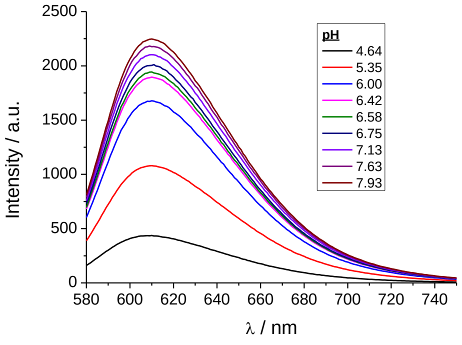

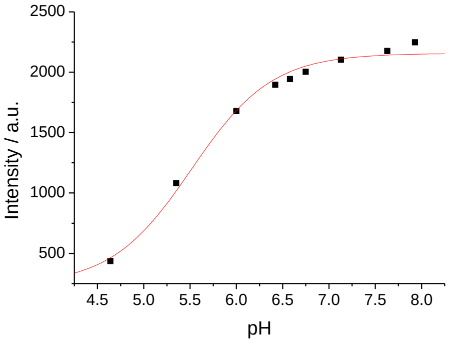

S94V

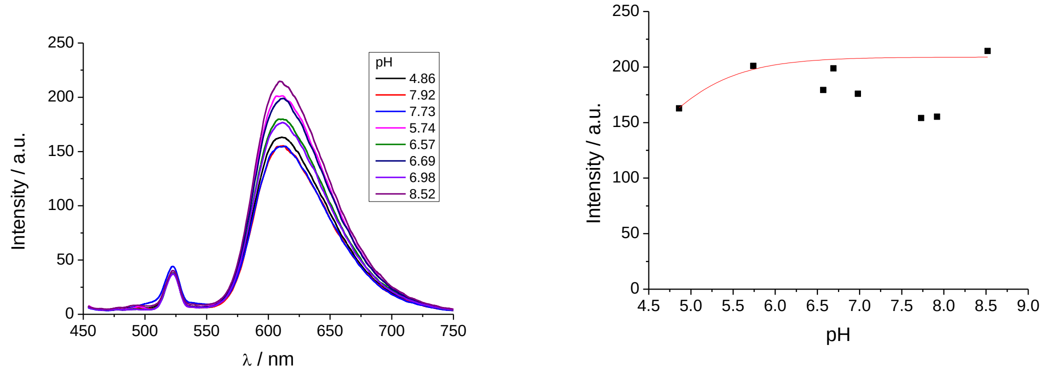

R205Y

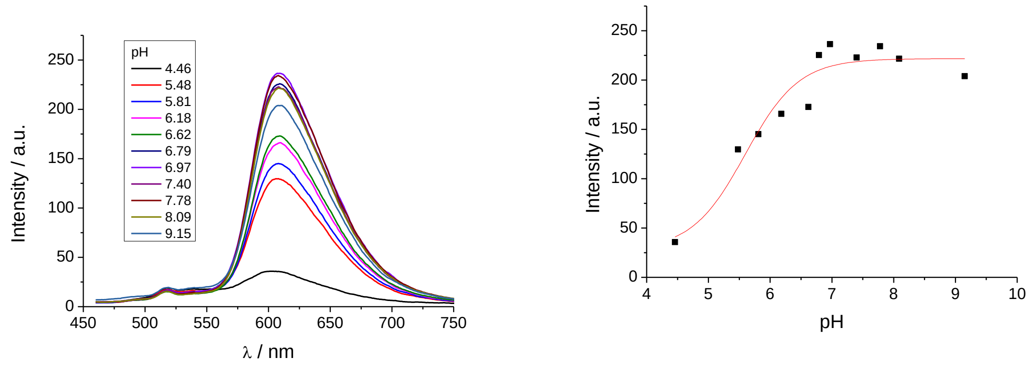

S94V-D162S

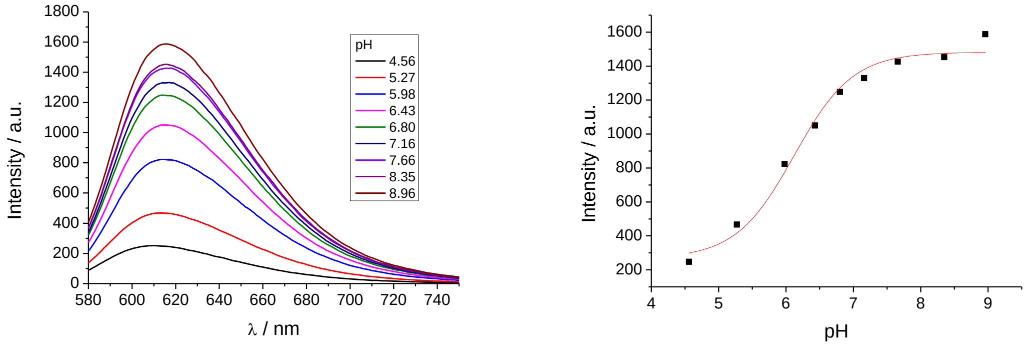

S94V-R205Y

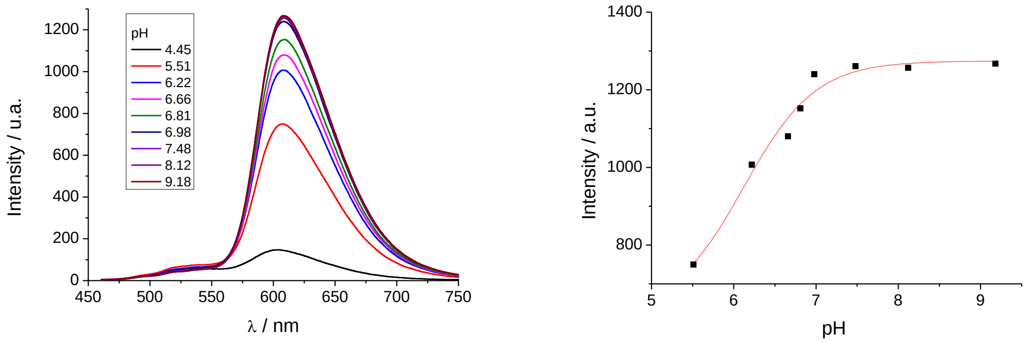

162S-R205Y

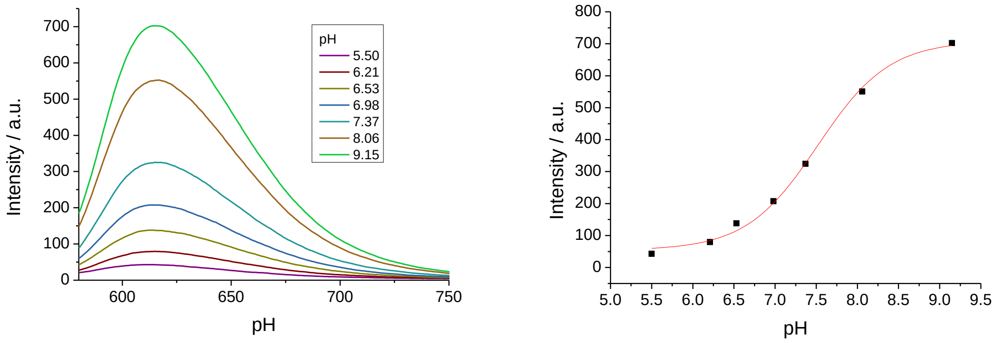

S94V-D162S-R205Y

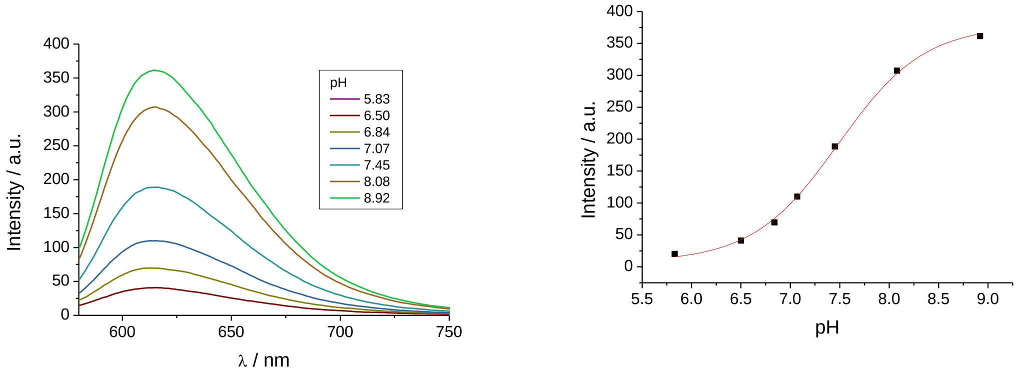

Figure S2. *pH dependence of the fluorescence intensity of all the mutants studied in this work.* (Left) Fluorescence emission spectra of all mutants studied in this work at different pH values. (Right) Maxima intensity values from emission spectra. The fitting curve was drawn using equation 1 in Methods.

mBeRFP

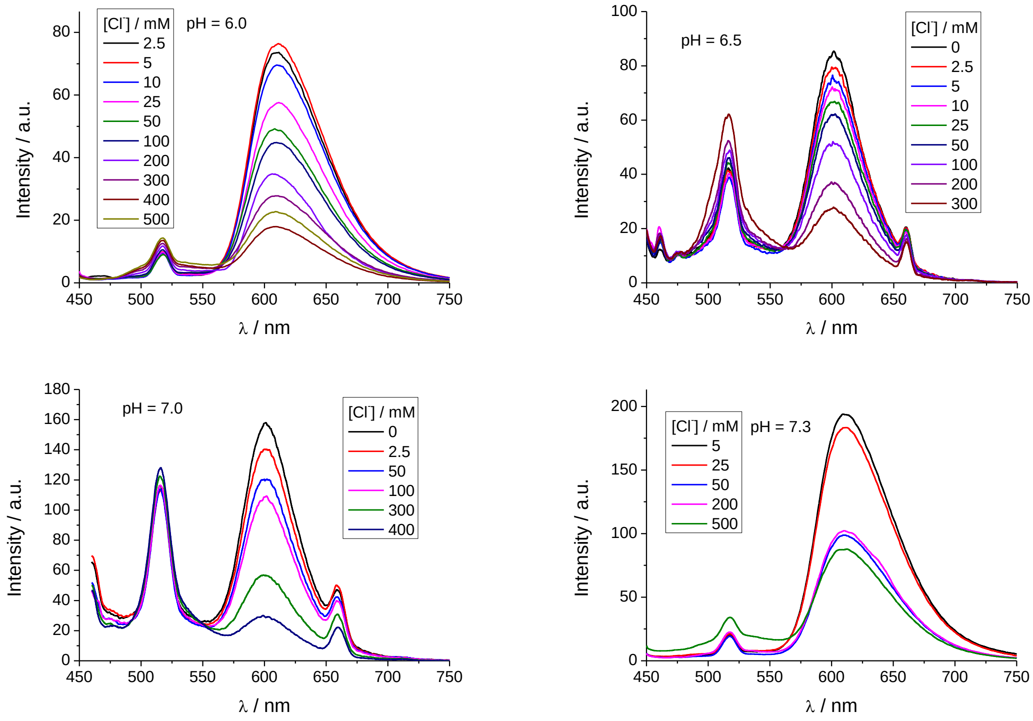

D162S

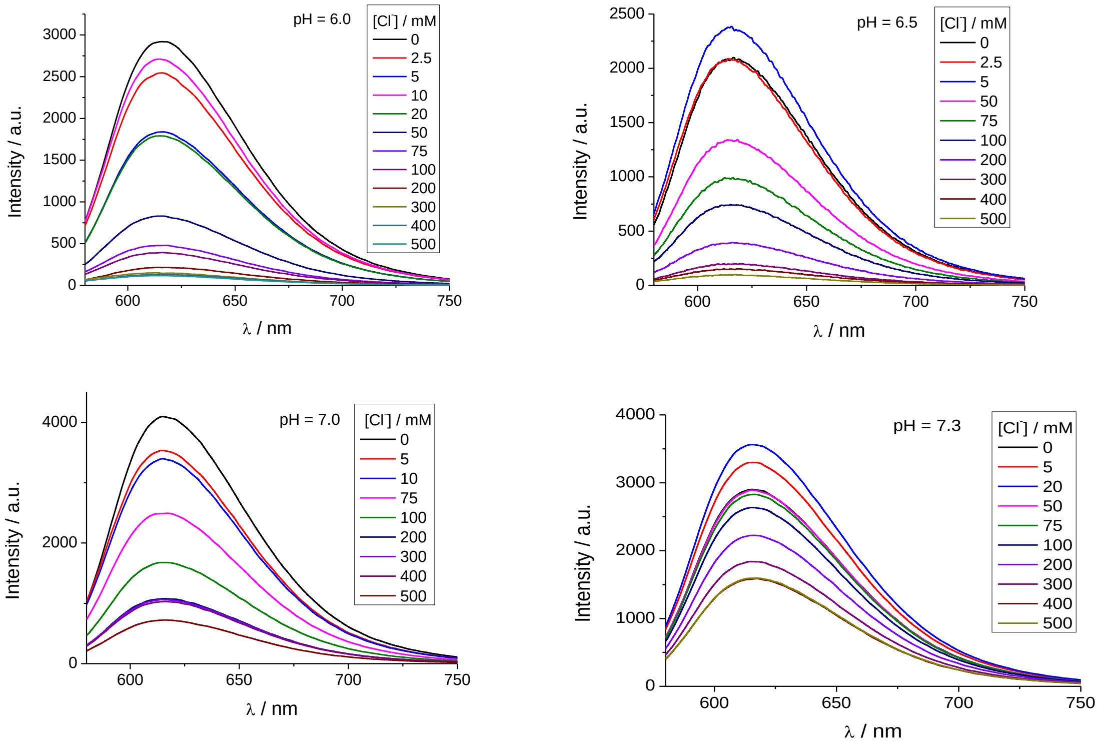

D162A

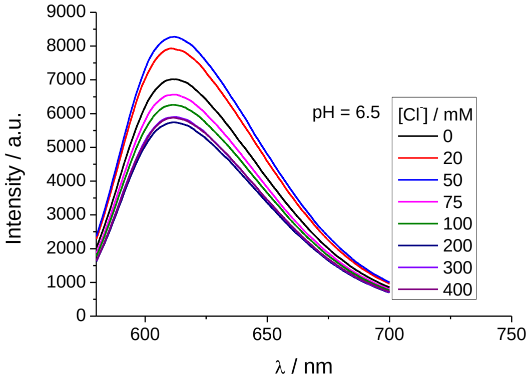

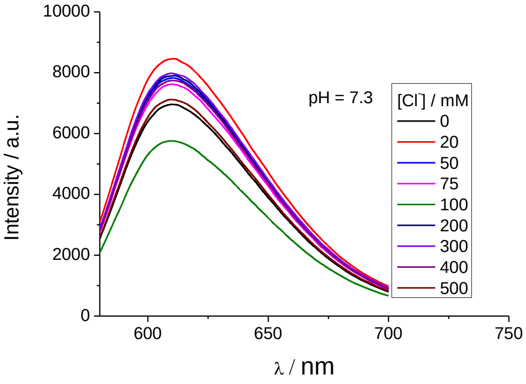

D162T

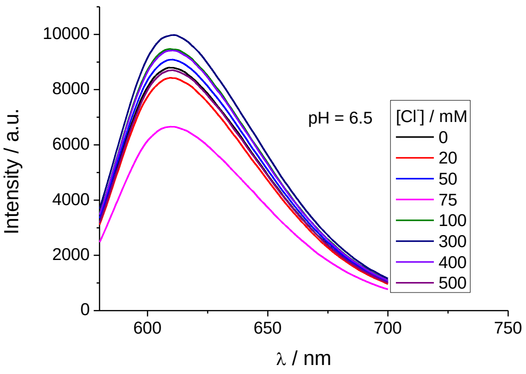

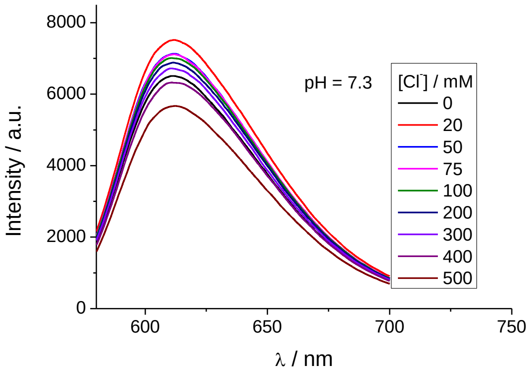

S94V

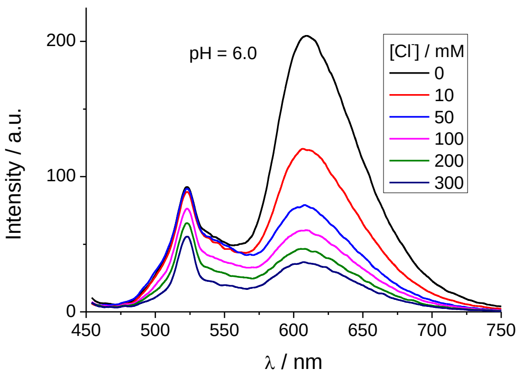

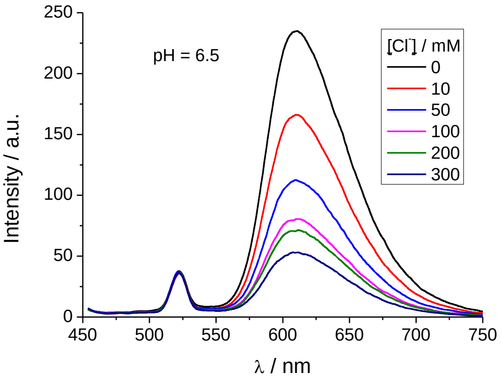

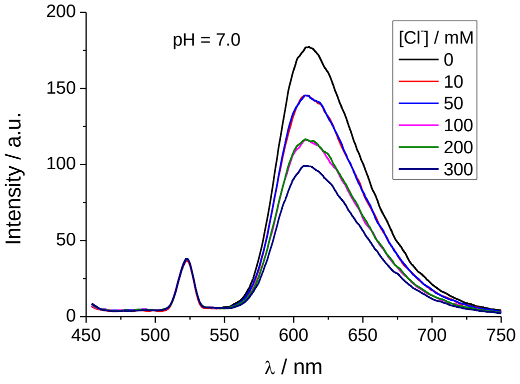

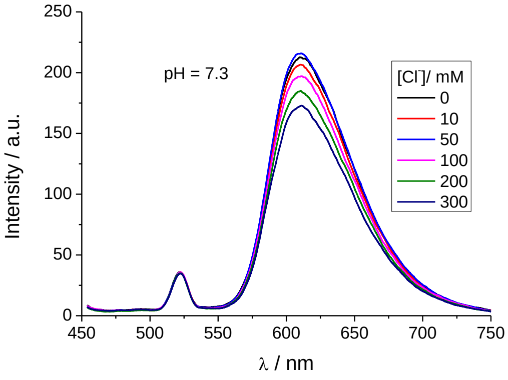

R205Y

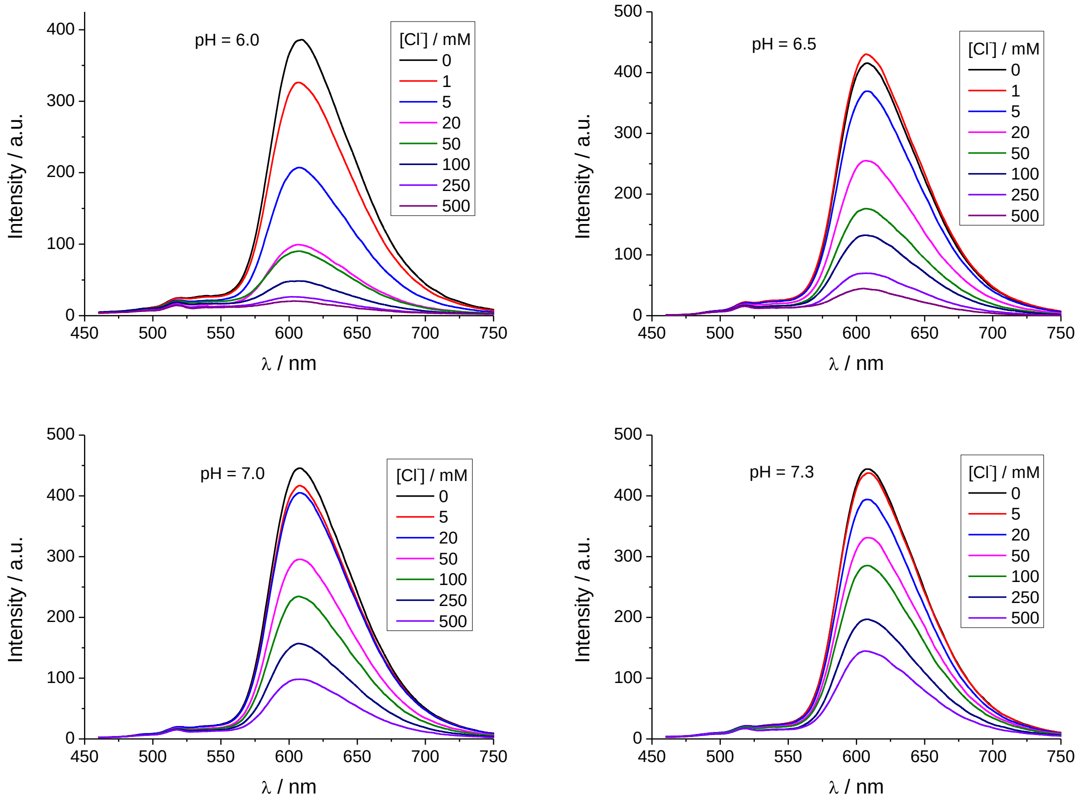

S94V-D162S

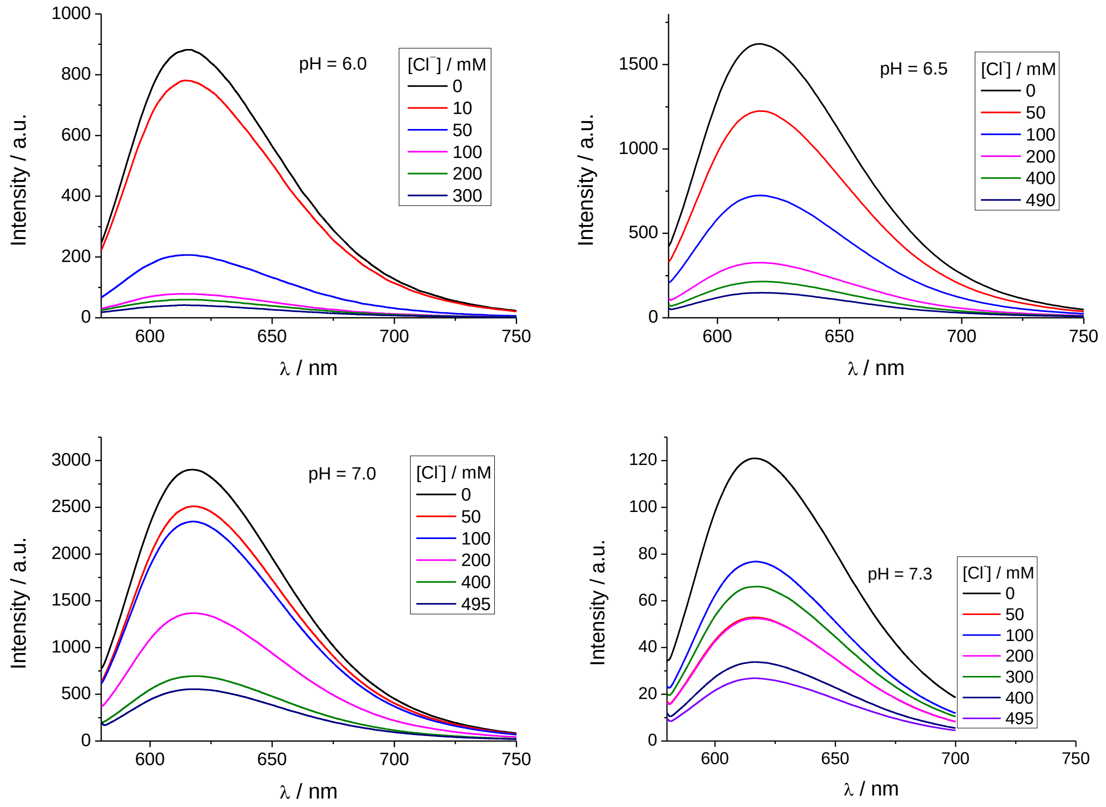

S94V-R205Y

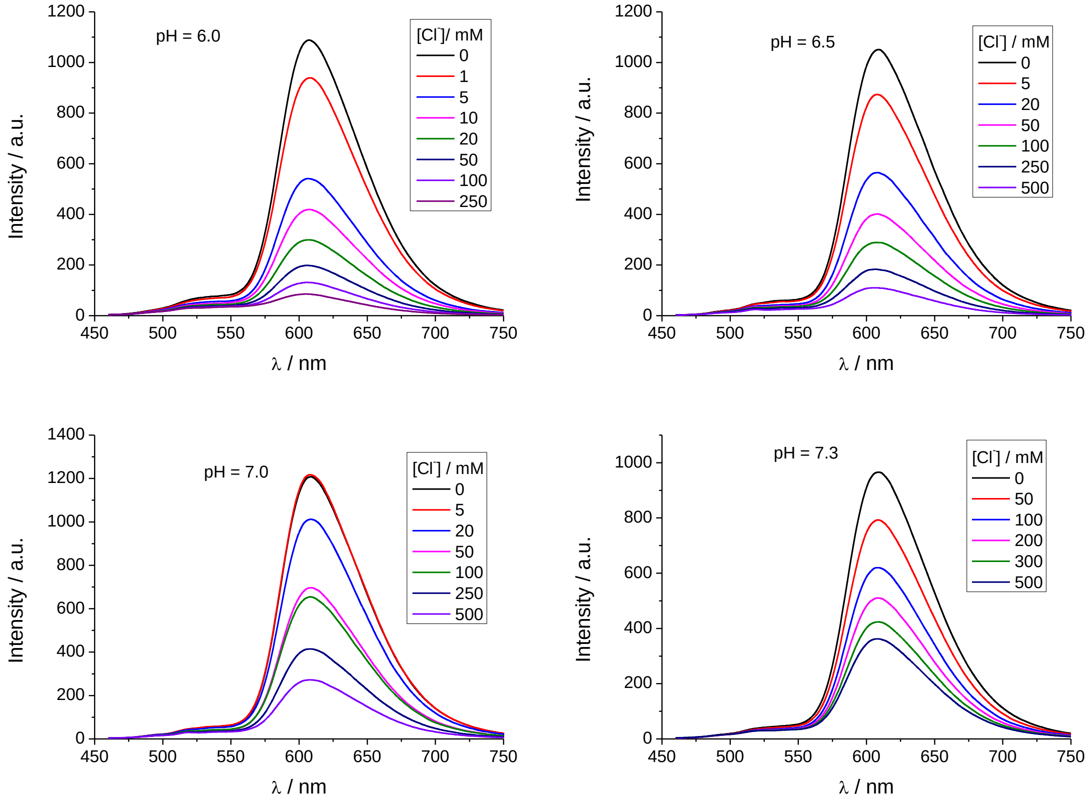

D162S-R205Y

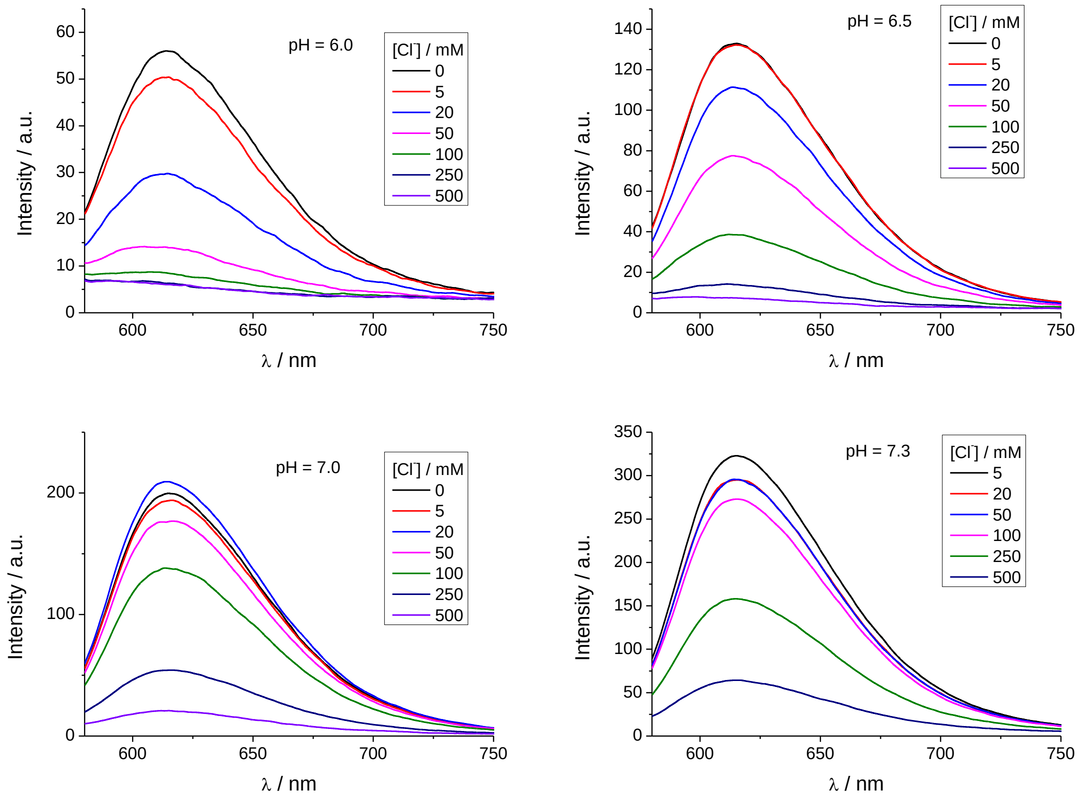

S94V-D162S-R205Y

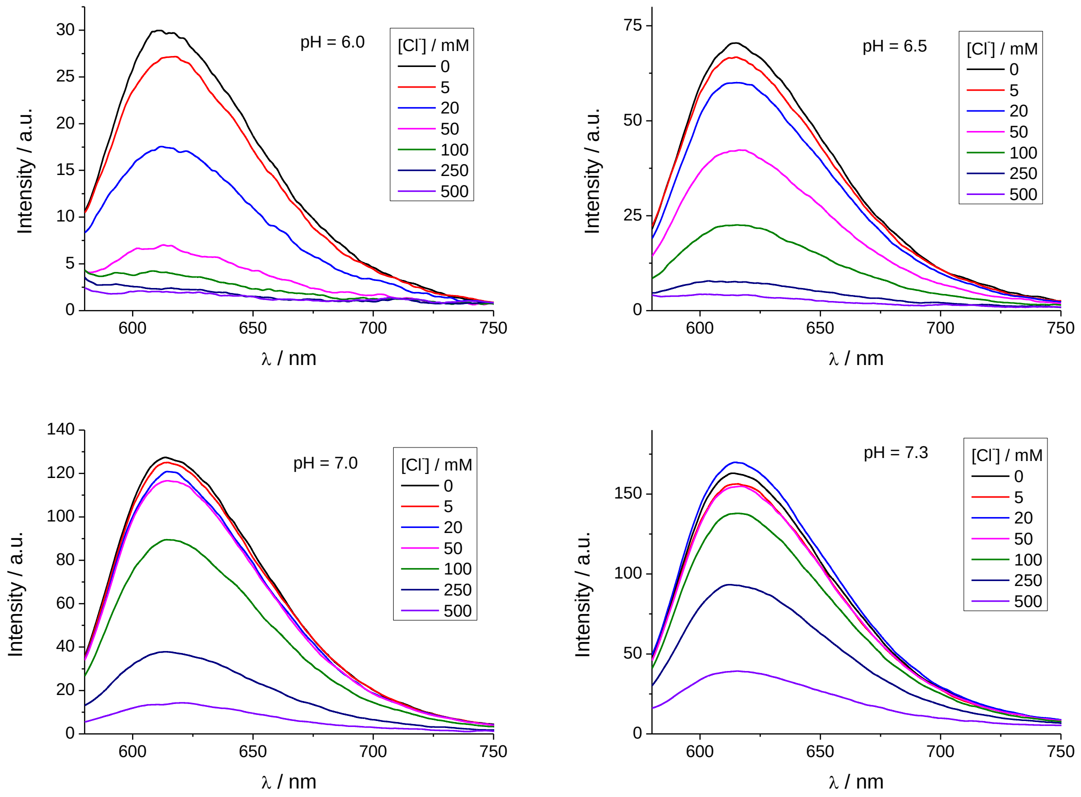

Figure S3. *Chloride titration at different pH values.* Fluorescence emission spectra of all mutants studied in this work at different chloride concentrations and four pH values.

mBeRFP

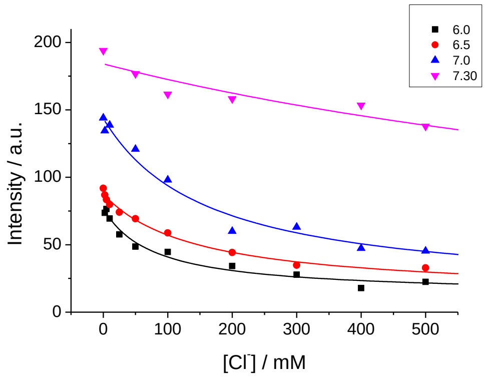

D162S

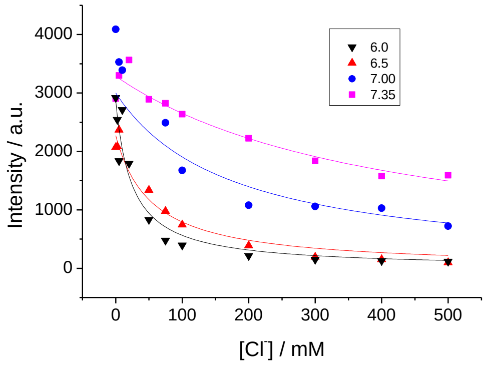

D162A

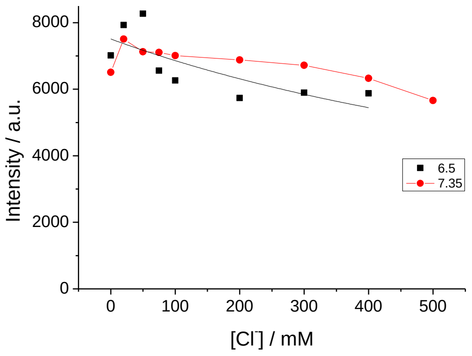

D162T

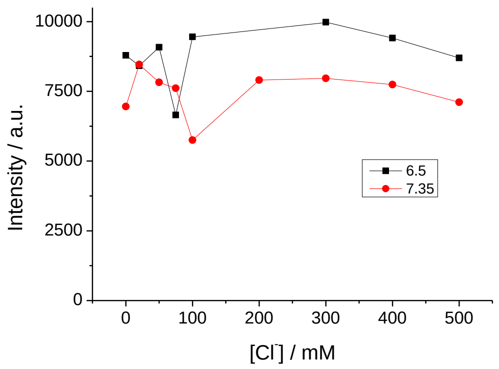

S94V

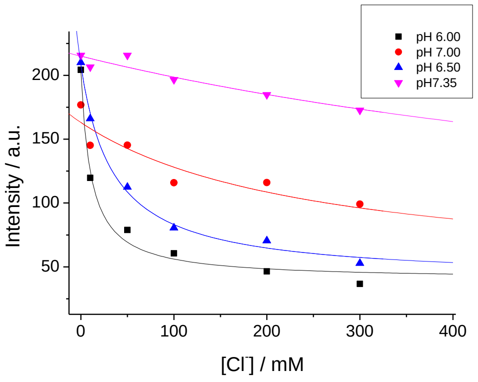

R205Y

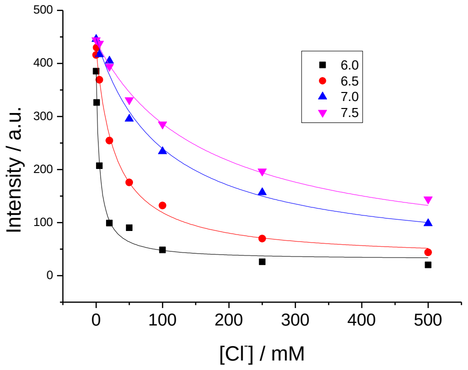

D162S-S94V

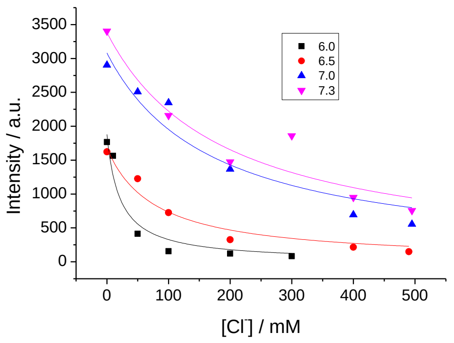

S94V-R205Y

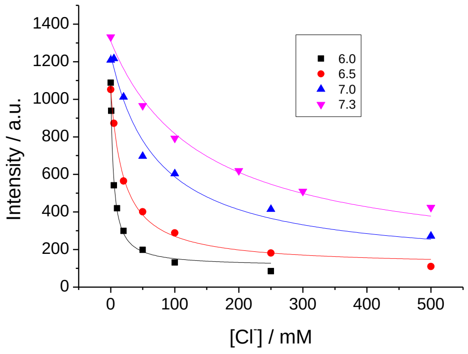

D162S-R205Y **D162S-S94V-R205Y**

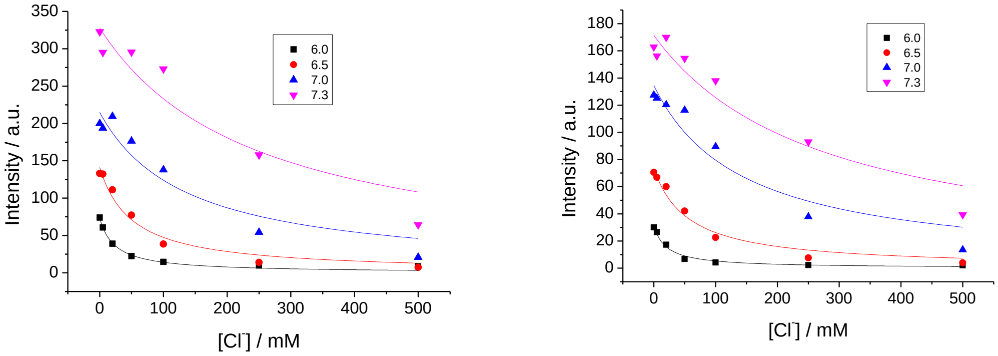

Figure S4. *Chloride titration at four different pH values. Fitting curves.* Maxima intensity values from emission spectra at different chloride concentrations and four different pH values. The fitting curves were drawn using equation 2 in Methods.

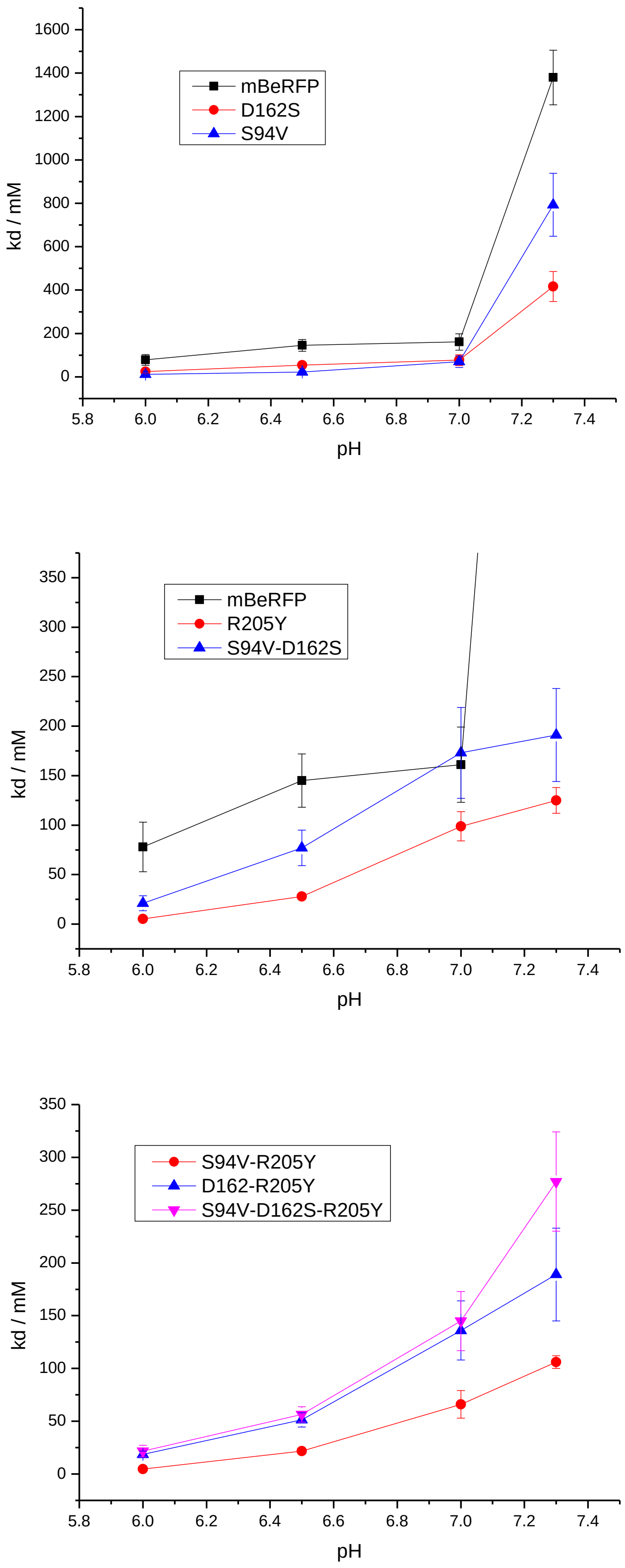

Figure S5. *pH dependence of the dissociation constant.* Chloride dissociation constant of all the mutants studied in this work determined at four different pH values.

Table S3. *Relative quantum yields and brightnesses*. The relative QYs and relative molar extinction coefficient were obtained using mBeRFP as a reference (Molar extinction coefficient = 65000 $M^{-1} cm^{-1}$). Relative molecular brightness is the product of the relative quantum yield and relative molar extinction coefficient.

	**Relative QY***	**Relative molar extinction coefficient***	**Molar extinction coefficient at maximum absorption wavelength ($M^{-1} cm^{-1}$)**	**Relative molecular brightness with respect mBeRFP**
D162S	4.7	0.198	12900	0.933
S94V	1.4	0.157	10200	0.220
R205Y	3.8	0.194	12580	0.735
S94V-D162S	0.7	0.354	23040	0.248
S94V-R205Y	0.85	0.182	11840	0.155
D162S-R205Y	11	0.027	1750	0.296
S94V-D162S-R205Y	3.4	0.042	2730	0.143

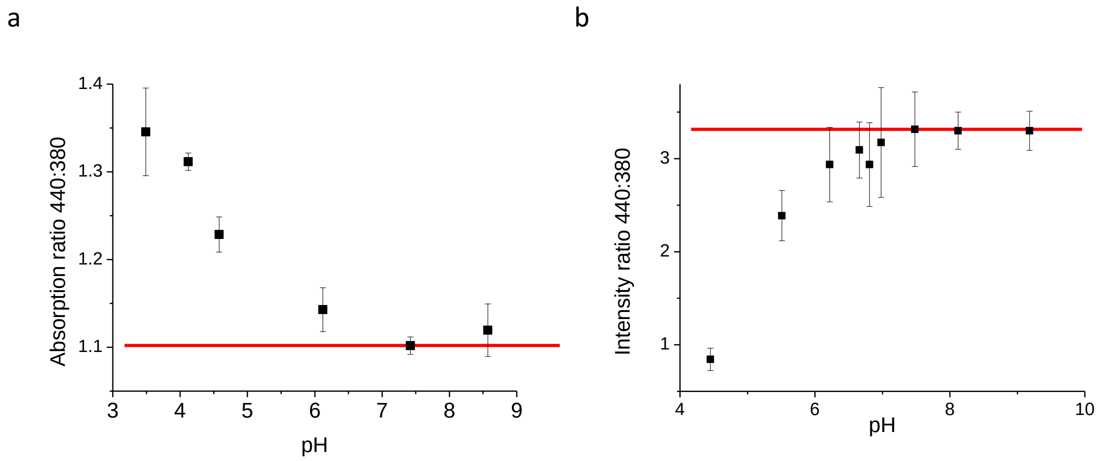

Figure S6. *Cis/trans isomerization in the (**a**) absorption and (**b**) excitation ratio values.* The line represents a visual to help identify the presence of only one isomer. Error bars represent the standard error from three independent measurements.

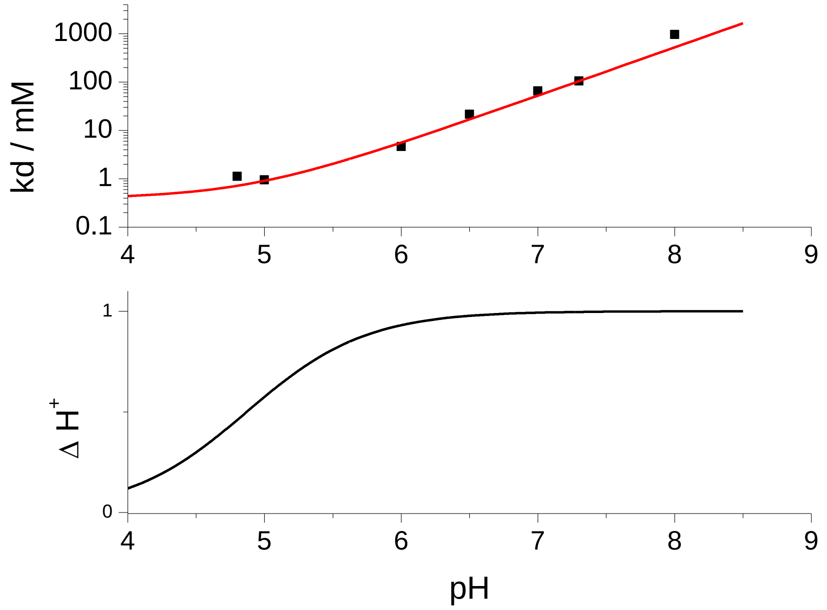

Figure S7. *Linkage between H^+ and Cl^- binding to mBeRFP S94V-R205Y.* The linkage is represented as the change in the logarithm of k_d and the net number of H^+ exchanged upon Cl^- binding derived from the fitting of k_d in the function of pH. The net number is expressed as follows: $\Delta H^+ = \delta ln\frac{1}{k_d}/\delta pH$

a

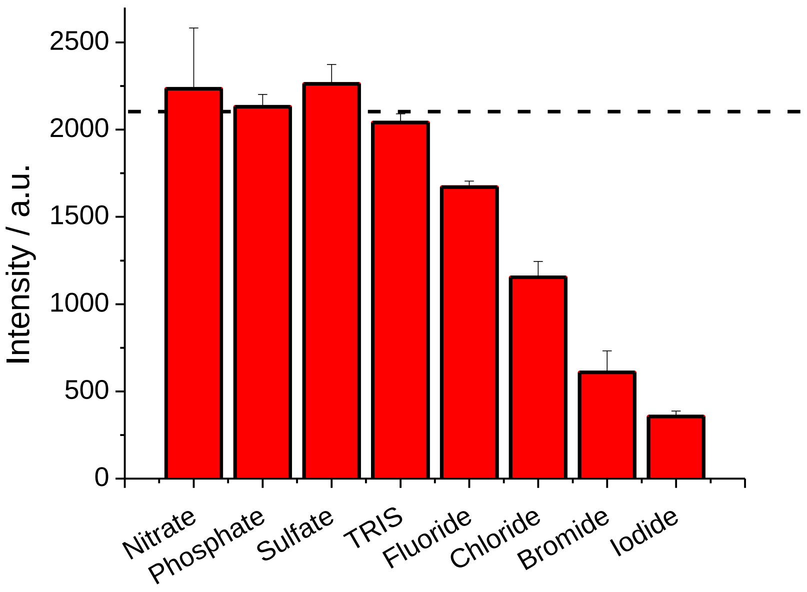

b

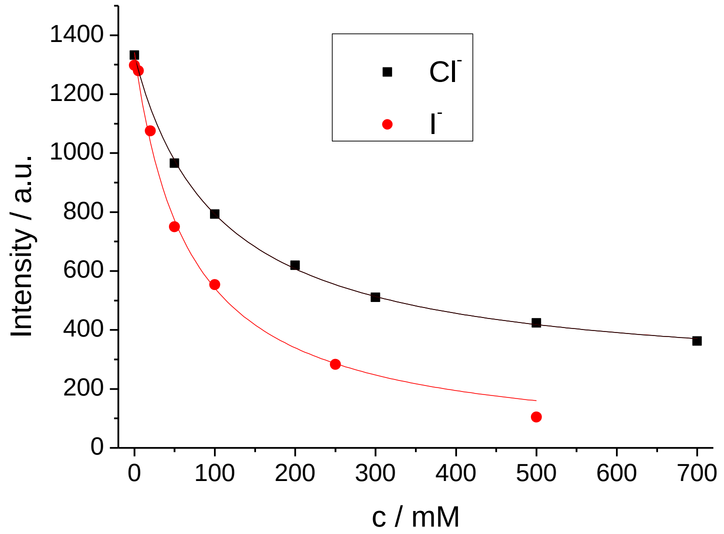

Figure S8. *mBeRFP S94V-R205Y anion dependence.* (**a**) Measurements of the effect of different anions at a concentration of 500 mM and pH = 7.30 on the intensity of *mBeRFP S94V-R205Y* (**b**) Comparison between chloride and iodide titration at pH 7.35. The curves are the fitting of equation 2 (see methods). $K_d^{Cl^-}$ = 106 ± 6 mM and $K_d^{I^-}$ = 68 ± 6 mM

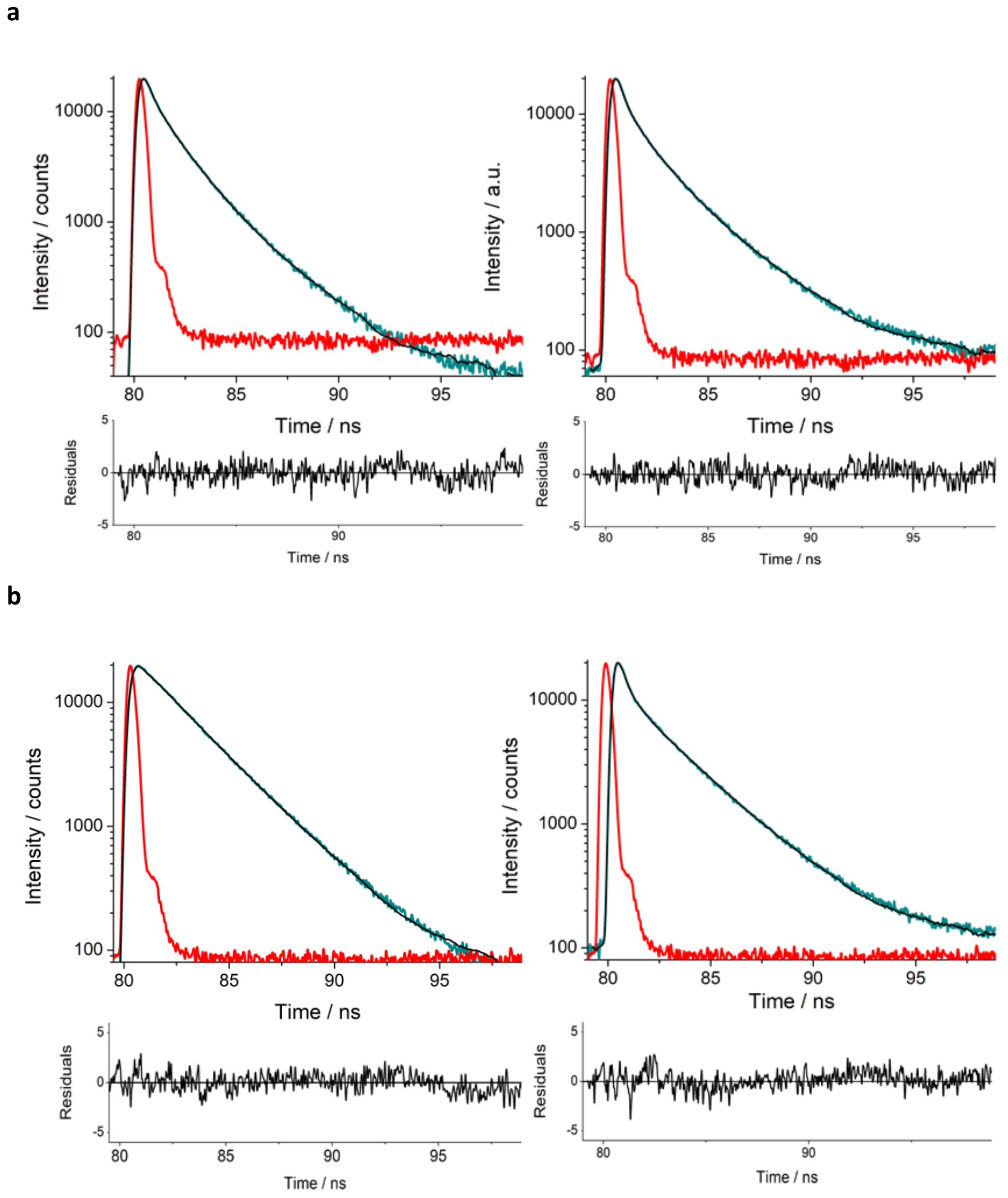

Figure S9. *Fluorescence decay histograms of mBeRFP S94V-R205Y* (in cyan). The samples were measured at pH = 7.3 and λ_{ex} = 375 nm. (**a**) λ_{em} = 505 nm at chloride concentrations of 0 (left) and 500 (right) mM. (**b**) λ_{em} = 610 nm at chloride concentrations of 0 (left) and 500 (right) mM. The instrument response function (in red), the fitting (black) and the residuals are also shown.

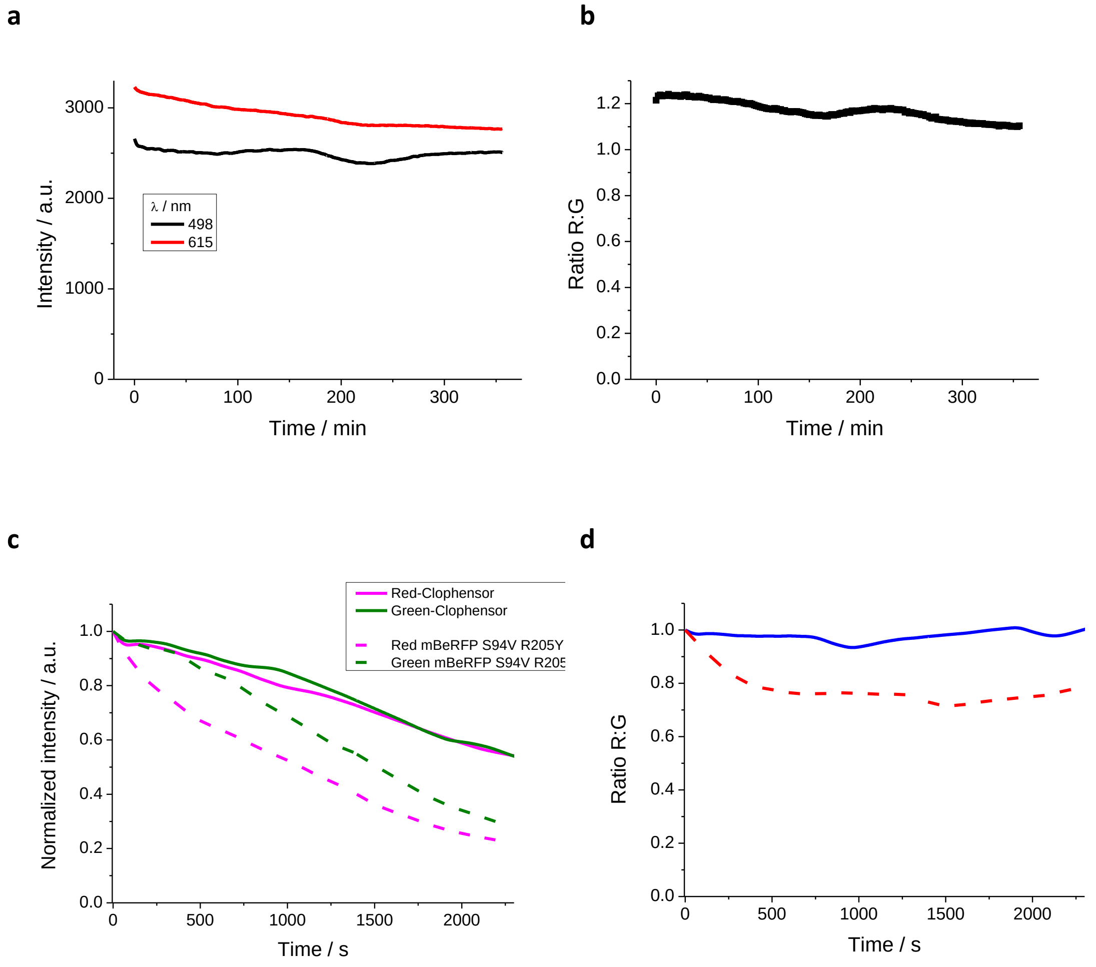

Figure S10. *mBeRFP S94V-R205Y photobleaching.* **(a)** Decrease in intensity at wavelengths of 498 and 615 nm in solution. (**b**) mBeRFP S94V-R205Y photobleaching effect on the fluorescence ratio in solution. (**c**) mBeRFP S94V-R205Y and ClopHensor photobleaching in HEK-293 cells. (**d**) ClopHensor and mBeRFP S94V-R205Y photobleaching effect on the fluorescence ratio in HEK-293 cells.

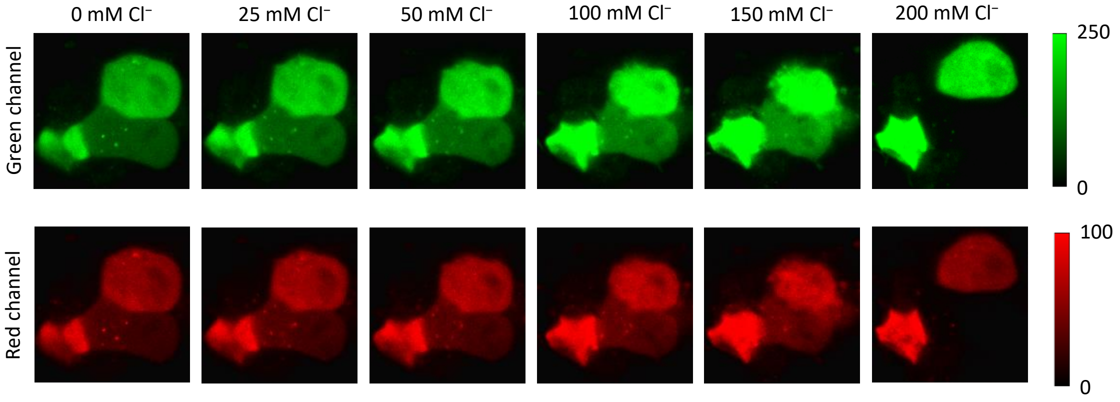

Figure S11. *Raw images of HEK-293 cells transfected with mBeRFP obtained using one photon-excitation microscopy.*

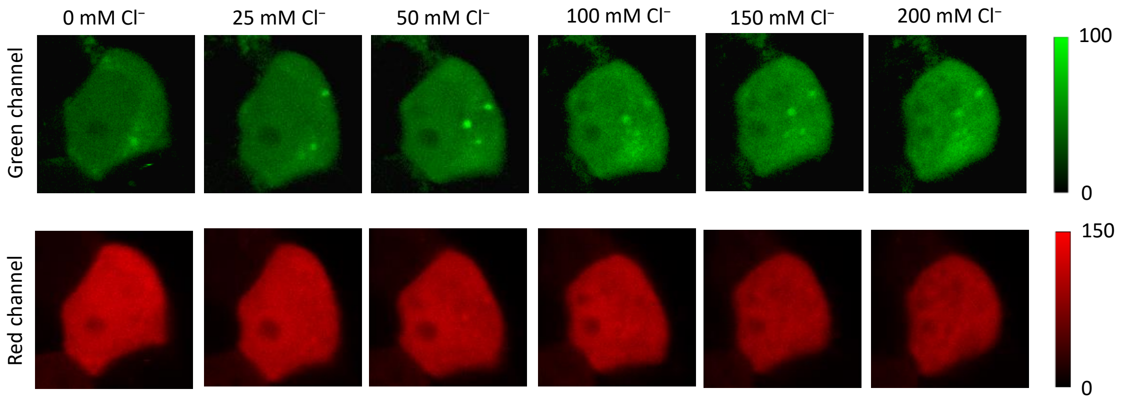

Figure S12. *Raw images of HEK-293 cells transfected with the S94V-R205Y mutant obtained using one-photon excitation microscopy.*

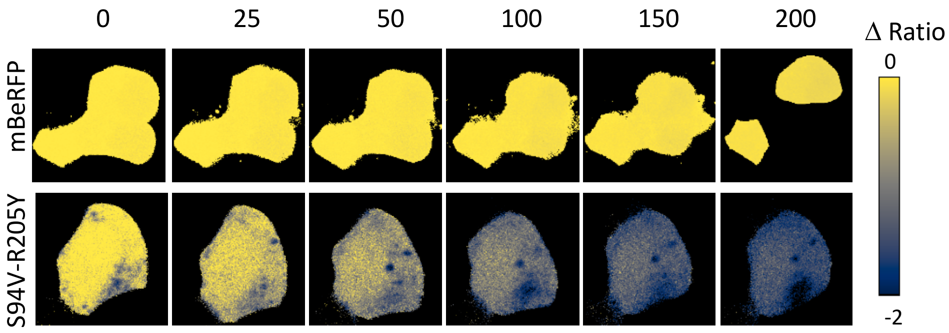

Figure S13. *Red/green ratio maps of HEK-293 cells clamped at different chloride concentrations.*

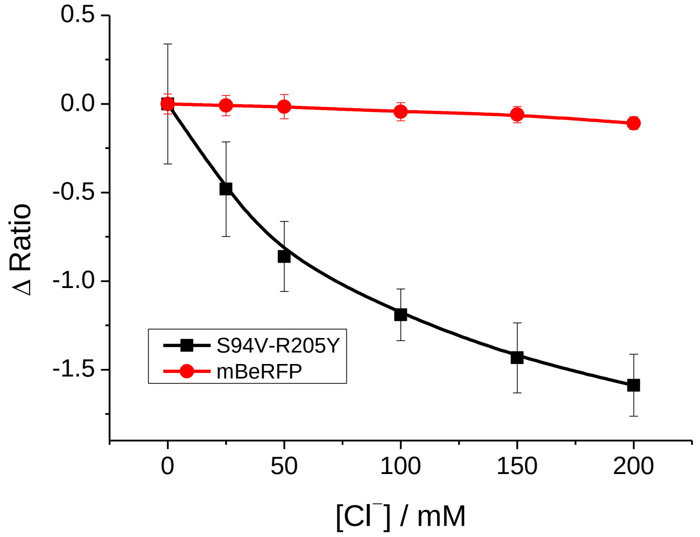

Figure S14. *Ratio changes in HEK-293 cells transfected with pmBeRFP or pmBeRFP S94V-R205Y and clamped at different chloride concentrations.*

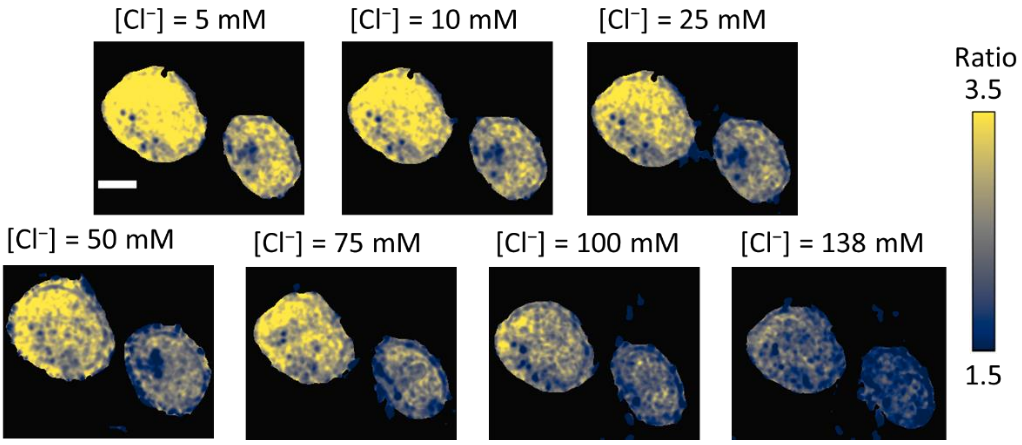

Figure S15. *Representative ratio maps of ClopHensor calibration.* The scale bar represents 10 µm.

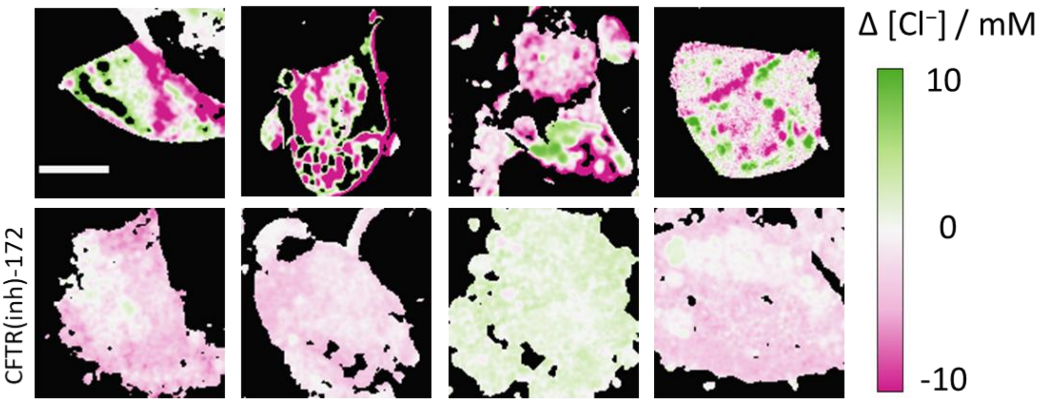

Figure S16. *Chloride changes in CaCo-2 cells transfected with pmBeRFP S94V-R205Y.* Images were obtained after the addition of 50 µM Br-cAMP to cells that were previously incubated in the absence (upper row) or presence (lower row) of the inhibitor CFTR(inh)-172 (10 µM) for 30 min. The scale bar represents 10 µm.

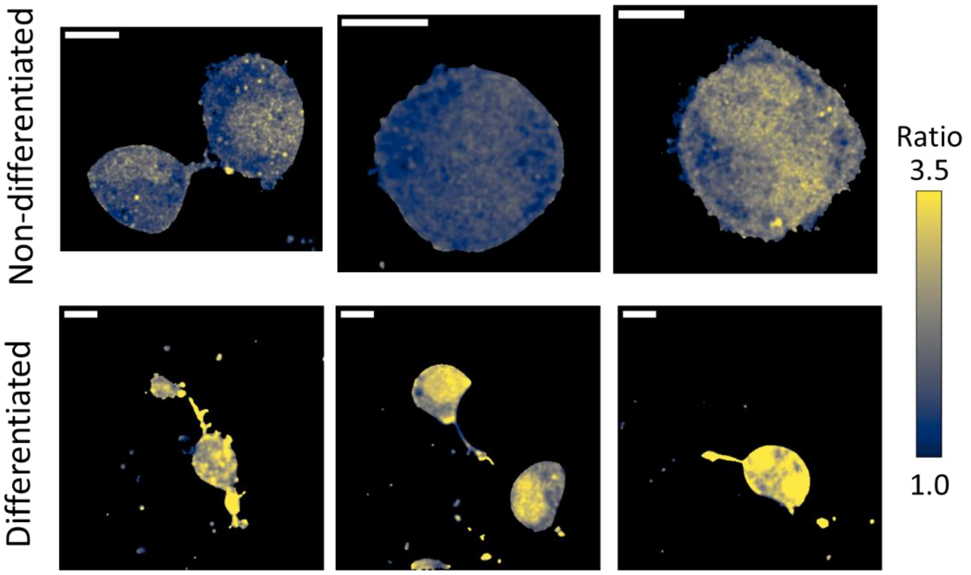

Figure S17. *Representative ratio maps of nondifferentiated (up) and differentiated (down) neuro 2a cells.* The scale bars represent 10 μm.

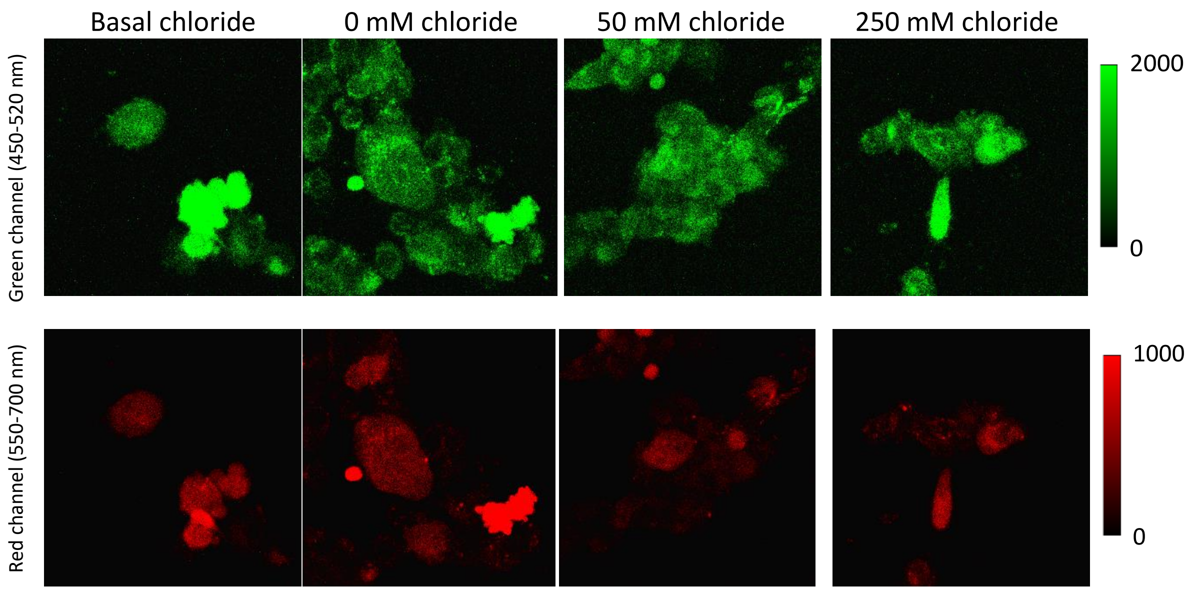

Figure S18. *Raw images of HEK-293 cells transfected with the S94V-R205Y mutant obtained using two-photon excitation microscopy*.

Supplementary Information Text

The ImageJ macro used to process and analyze the images obtained in this work.

```
run("Bin...", "x=3 y=3 bin=Sum"); //(only for two photon
excitation)
run("Gaussian Blur...", "sigma=0.5");
selectWindow("Channel-0001");
rename("Green");
run("Green");
selectWindow("Channel-0002");
rename("Red");
run("Red");
run("Duplicate...", "title=ROI");
run("8-bit");
setAutoThreshold("Huang dark");
run("Make Binary");
run("Median...", "radius=3");
selectWindow("Green");
imageCalculator("Multiply", "Green","ROI");
imageCalculator("Divide", "Green","ROI");
run("Measure");
selectWindow("Red");
imageCalculator("Multiply", "Red","ROI");
imageCalculator("Divide", "Red","ROI");
run("Measure");
imageCalculator("Divide create", "Red","Green");
selectWindow("Result of Red");
rename("Ratio");
run("Measure");
```

Movie S1 (separate file). Red/green ratio maps of HEK-293 cells transfected with mBeRFP or pmBeRFP S94V-R205Y and clamped at different chloride concentrations. The figure shows the changes in the ratio values in HEK-293 cells transfected with pmBeRFP or pmBeRFP S94V-R205Y and clamped at different chloride concentrations.
